# LAMC2 marks a tumor-initiating cell population with an aggressive signature in pancreatic cancer

**DOI:** 10.1186/s13046-022-02516-w

**Published:** 2022-10-26

**Authors:** Donatella Delle Cave, Silvia Buonaiuto, Bruno Sainz, Marco Fantuz, Maria Mangini, Alessandro Carrer, Annalisa Di Domenico, Tea Teresa Iavazzo, Gennaro Andolfi, Carme Cortina, Marta Sevillano, Christopher Heeschen, Vincenza Colonna, Marco Corona, Antonio Cucciardi, Martina Di Guida, Eduard Batlle, Annachiara De Luca, Enza Lonardo

**Affiliations:** 1 Institute of Genetics and Biophysics “A. Buzzati-Traverso”, National Research Council (CNR-IGB), 80131 Naples, Italy; 2 Department of Cancer Biology, Instituto de Investigaciones Biomedicas “Alberto Sols” (IIBM), CSIC-UAM, 28029 Madrid, Spain; 3 Chronic Diseases and Cancer, Area 3-Instituto Ramon Y Cajal de Investigacion Sanitaria (IRYCIS), 28034 Madrid, Spain; 4 Centro de Investigación Biomédica en Red, Área Cáncer, CIBERONC, ISCIII, 28029 Madrid, Spain; 5 Department of Biology, University of Padova, 35129 Padova, Italy; 6 Veneto Institute of Molecular Medicine (VIMM), 35129 Padova, Italy; 7 Institute for Experimental Endocrinology and Oncology, “G. Salvatore” (IEOS), Second Unit, Consiglio Nazionale Delle Ricerche (CNR), 801310 Naples, Italy; 8 Institute for Research in Biomedicine (IRB Barcelona), Barcelona Institute of Science and Technology (BIST), Baldiri Reixac 10, 08028 Barcelona, Spain; 9 Centro de Investigación Biomédica en Red de Cáncer (CIBERONC), 08028 Barcelona, Spain; 10 State Key Laboratory of Oncogenes and Related Genes, Center for Single-Cell Omics, School of Public Health, Shanghai Jiao Tong University School of Medicine, Shanghai, China

**Keywords:** Pancreatic ductal adenocarcinoma (PDAC), Laminin γ2 (LAMC2), Tumor-initiating cells (TICs), TGF-β signaling, Vactosertib

## Abstract

**Background:**

Tumor-initiating cells (TIC), also known as cancer stem cells, are considered a specific subpopulation of cells necessary for cancer initiation and metastasis; however, the mechanisms by which they acquire metastatic traits are not well understood.

**Methods:**

*LAMC2* transcriptional levels were evaluated using publicly available transcriptome data sets, and LAMC2 immunohistochemistry was performed using a tissue microarray composed of PDAC and normal pancreas tissues. Silencing and tracing of *LAMC2* was performed using lentiviral shRNA constructs and CRISPR/Cas9-mediated homologous recombination, respectively. The contribution of LAMC2 to PDAC tumorigenicity was explored *in vitro* by tumor cell invasion, migration, sphere-forming and organoids assays, and *in vivo* by tumor growth and metastatic assays. mRNA sequencing was performed to identify key cellular pathways upregulated in LAMC2 expressing cells. Metastatic spreading induced by LAMC2- expressing cells was blocked by pharmacological inhibition of transforming growth factor beta (TGF-β) signaling.

**Results:**

We report a LAMC2-expressing cell population, which is endowed with enhanced self-renewal capacity, and is sufficient for tumor initiation and differentiation, and drives metastasis. mRNA profiling of these cells indicates a prominent squamous signature, and differentially activated pathways critical for tumor growth and metastasis, including deregulation of the TGF-β signaling pathway. Treatment with Vactosertib, a new small molecule inhibitor of the TGF-β type I receptor (activin receptor-like kinase-5, ALK5), completely abrogated lung metastasis, primarily originating from LAMC2-expressing cells.

**Conclusions:**

We have identified a highly metastatic subpopulation of TICs marked by LAMC2. Strategies aimed at targeting the LAMC2 population may be effective in reducing tumor aggressiveness in PDAC patients. Our results prompt further study of this TIC population in pancreatic cancer and exploration as a potential therapeutic target and/or biomarker.

**Supplementary Information:**

The online version contains supplementary material available at 10.1186/s13046-022-02516-w.

## Background

Pancreatic ductal adenocarcinoma (PDAC) is the most common histologic subtype of pancreatic cancer. The disease ranks as the fourth leading cause of cancer-related deaths worldwide, with a dismal 5-year survival rate of less than 5% [1]. PDAC is a devastating disease due to late diagnosis, its aggressive nature (early metastasis), lack of effective treatment options and a limited understanding of the cellular and molecular mechanisms underlying its initiation and progression. Furthermore, chemotherapy resistance and tumor recurrence are two unresolved clinical problems associated with PDAC treatment [2].

Many cancers including PDAC are hierarchically organized and made up of functionally heterogeneous population of cells. Convincing evidence now demonstrates the critical relevance of the tumor-initiating cell (TIC) or cancer stem cell (CSC) compartment in PDAC heterogeneity and aggressiveness [3]. TICs share several features with normal stem cells, such as self-renewal and differentiation potential [4, 5], and TICs also play critical roles in tumor progression and metastasis [6, 7]. However, from a clinical perspective, targeting TICs is difficult as TICs are highly refractory to conventional therapies, a feature that has been show to drive tumor relapse [8].

These data suggest that we need to reconsider our approach for treating cancer, as we should also take into consideration the successful elimination of TICs. Thus, there is an urgent need to better understand the heterogeneous TICs subpopulations in order to develop more effective therapies against them [9]. While several TIC markers have been proposed for PDAC [6, 10–17], no one marker can accurately identify all TICs nor capture the diverse heterogeneity that exists within the TIC population. Consequently, it is necessary to further study and characterize the TIC (sub-)populations in hopes of discovering new markers that can better define these cells. Herein, we identify and characterize a TIC population in PDAC marked by high expression levels of Laminin γ2 (LAMC2). LAMC2 is a subunit of the heterotrimeric glycoprotein laminin-332, which is a fundamental component of epithelial basement membranes and regulates cell motility and adhesion [18]. Interestingly, high LAMC2 expression correlates with poorer survival [19].

Using genetic CRISPR-Cas9 targeting, we show that LAMC2-high cells, compared with their CRISPR-edited LAMC2-low counterparts, display enhanced sphere-forming and *in vivo* tumorigenic capacities, enhanced migratory and invasive potentials and apparent chemoresistance. RNA sequencing of LAMC2-high-generated tumors showed increased transforming growth factor beta (TGF-β) pathway activity and an aggressive squamous phenotype compared to the LAMC2-low-derived tumors. Specifically, we found that TGF-β/Smad signaling is essential for LAMC2-mediated maintenance of TIC features, and that LAMC2-low or LAMC2 KD (Knock-Down) reduced TGF-β/Smad signaling. Intriguingly, targeting the tumor bulk cells as well as LAMC2-high TICs with Vactosertib, a new specific inhibitor of the TGF-β receptor type 1 (TGFBR1), efficiently inhibited PDAC progression and abrogated metastasis. Therefore, this novel tumorigenic and metastatic LAMC2-high population in PDAC represents a promising new target for the development of more effective therapeutic strategies against this deadly disease.

## Methods

### PDAC cultures

Tumor-derived primary cell lines #215 and #253 (Tissue derivation: primary pancreatic tumor; Carcinoma type: pancreatic ductal adenocarcinoma) were cultured in RPMI, 10% FBS, and 50 units/ml penicillin/streptomycin [4]. The human PDAC cancer cell lines L3.6pl (Tissue derivation: metastatic lymph node; Carcinoma type: adenosquamous carcinoma), PANC-1 (Tissue derivation: pancreatic tumor; Carcinoma type: ductal carcinoma) and immortalized HPNE cells were maintained as previously described [4]. Their identity (annually) and *Mycoplasma* free-state (bi-weekly) were tested by DNA fingerprinting using short tandem repeat (STR) profiling and by PCR-based, MycoAlert Mycoplasma Detection Kit (Lonza, Bioscience), respectively. Each cell line was used for 4/5 passages post thawing.

### RNA Preparation and Real-Time quantitative PCR

Total RNA was extracted with the Eurogold TRIFAST kit (Euroclone) according to the manufacturer’s instructions. One microgram of total RNA was used for cDNA synthesis with High-Capacity reverse transcriptase (Thermofisher). Quantitative real-time PCR was performed using a SYBR Green PCR master mix (Thermofisher), according to the manufacturer’s instructions. The list of primers utilized are listed in Table 1. *n* $\ge 3.$ Statistical significance was assessed by Student's t-test.

**Table 1:** Primers used for Real-Time quantitative PCR

**Gene symbol**	**Forward primer (5'- > 3')**	**Reverse primer (5'- > 3')**
***CDH1***	TGCCCAGAAAATGAAAAAGG	GGATGACACAGCGTGAGAGA
***CD44***	CACGTGGAATACACCTGCAA	GACAAGTTTTGGTGGCACG
***CD133***	GCAATCTCCCTGTTGGTGAT	TCAGATCTGTGAACGCCTTG
***GAPDH***	CAGGAGCGAGATCCCT	GGTGCTAAGCAGTTGGT
***VIM***	CTCAATGTCAAGGGCCATCT	TGCCCTTAAAGGAACCAATG
***LAMC2***	GGAAAGGAAGGAGCTGGAGT	TGTTGATCTGGGTCTTGGCT
***CNT1***	TCCAGAGCGTCAATCCAGAG	ACTCTCTGCACACTCACTCC
***CNT2***	GTTTCTGCAAAACACACGCC	ACACCCATTTCGTCCAAAGC
***CNT3***	GCCGATCGTGGTTTTCTTCA	GTCATGATGGCGTGGAGTTC
***SNAIL1***	CTTCCAGCAGCCCTACGAC	CGGTGGGGTTGAGGATCT
***MMP2***	ATAACCTGGATGCCGTCGT	AGGCACCCTTGAAGAAGTAGC
***MMP10***	GGCTCTTTCACTCAGCCAAC	TCTCCCCTCAGAGTGCTGAT

### Protein isolation and western blot analysis

Cells were lysed with RIPA buffer (50 mM Tris–HCl at pH 7.6, 150 mM NaCl, 1% NP-40, 0.5% sodium deoxycholate, 0.1% SDS, 5 mM EDTA plus proteases and phosphatases inhibitors) for 1 h at 4 °C. Total protein quantification was performed with Bio-Rad Protein Assay Dye Reagent concentrate. A total of 40 μg of protein was separated on 15% SDS–PAGE gels at 100 V and transferred to PVDF membranes for 2 h at 200 mA. PVDF membranes were hybridized with antibodies against LAMC2 (AMAB 91,098, Sigma-Aldrich), SMAD2 (cell signaling – 5339), pSMAD2 (cell signaling – 3108S), GAPDH (cell signaling – 2118), β-actin (E-AB-20058, Elabscience), MMP-2 (4022S, Cell Signaling), Vimentin (3932, Cell Signaling) and α-tubulin (2144, Cell Signaling) and subsequently hybridized with peroxidase-conjugated goat anti-mouse or anti-rabbit Ig secondary antibodies (DPVR-HRP, Immunologic), and then visualized by enhanced chemiluminescence (ECL Nova 2.0 XLS071, 2050 Cyanagen). *n* $\ge 3.$

### Lentiviral shRNA delivery

The lentiviral shuttle backbone pLKO shRNA plasmid was used (Mission SIGMA). As a control we used the pLKO shRNA empty expression vectors. Cells were transduced with lentiviral particles in the presence of polybrene (8 µg/ml, Sigma). Briefly, cells were seeded at a density of 30,000 cells per well in 24 multi-well plates and allowed to adhere overnight. The following day, the cells were infected with lentiviral particles for 6 h. Stably transduced cells were obtained by selection with puromycin (500 µg/ml).

### Flow cytometry and cell sorting

Flow cytometry analysis or fluorescence-activated cell sorting (FACS) was performed using anti-human CD133-APC (BioLegend – 372805), TGFβR1-Alk5 (cell signaling—3712) and anti-human CD44-APC (BioLegend – 338805) to identify CSCs. 7AAD (BD) was used for exclusion of dead cells. Samples were analyzed by flow cytometry using a FACS Canto II (BD) and data were analyzed with FlowJo 9.2 software (Ashland, OR). Experiments were repeated a minimum of three independent times, with triplicate samples. Statistical significance was assessed by Student's t-test.

### Immunofluorescence

Cells were fixed in 4% paraformaldehyde (PFA) for 20 min at room temperature. After blocking with 5% bovine serum albumin in PBS-Triton 0.1%, cells were incubated with unconjugated primary antibody: LAMC2 (SIGMA, HPA024638-100U), pSMAD2 (cell signaling – 3108S) overnight at 4 °C in the dark, and 24 h later counterstained with a fluorescent secondary antibody. The nuclei of cells were stained with DAPI (SIGMA). Images were acquired at room temperature using the LEICA DMI6000 inverted microscope (Leica, Heidelberg, Germany) with a DC 350 FX camera (Leica). n $\ge 3.$

### Sphere-formation assay

Pancreatic cancer spheres were generated and expanded in CSCs media composed of: Advanced DMEM:F12 (GIBCO) supplemented with 1 × glutaMAX (GIBCO), 1 × B-27 (GIBCO), 1 × N2 (GIBCO), 20 ng/ml bFGF (basic fibroblast growth factor) (Invitrogen), and 50 ng/ml EGF (epidermal growth factor) (Peprotech, London, UK). Five hundred cells per 500 µl of sphere medium were seeded in 24 ultra-low attachment plates (Corning B.V., Schiphol-Rijk, Netherlands) as described previously [20]. After 7 days of incubation, spheres were typically > 75 µm large. For serial passaging, 7-day-old spheres were harvested using 40 µm cell strainers, dissociated into single cells with trypsin, and then regrown for an additional 7 days. Cultures were kept no longer than 4 weeks after thawing from frozen stocks (passage 3–4). Statistical significance was assessed by Student's t-test.

### Matrigel embedding culture assay

Five hundred PDAC cells were embedded in 50 µL of 100% BME2 (Cultrex) and seeded in 24 multi-well plates (Corning). The formed spheres, here termed organoid-like structures, were cultured in CSCs media for 7 days and counted using a light microscope. Statistical significance was assessed by Student's t-test.

### Migration assays

Migration assays were performed using Boyden chambers (Corning). Briefly, 25,000 tumor-derived primary cell lines #215 and #253 and PANC-1 (sh empty or sh*LAMC2)* were added to the inserts of the chamber for 22 h at 37 °C. Migrated cells were fixed in 4% PFA and stained with DAPI. The ratio of cells in the lower chamber versus total seeded cells was calculated. *n* $\ge 3.$ Statistical significance was assessed by Student's t-test.

### Wound healing assays

Cells were seeded at the appropriate number in 6-well culture plates until 100% confluence was reached (approximately 24 h). Then, confluent cultures were scratched using a 200 μL pipette tip and then incubated at 37 °C for 24 and 48 h. At the indicated times, images of the wounds were acquired using a LEICA DMI6000 inverted microscope (Leica, Heidelberg, Germany) with a DC 350 FX camera (Leica). The wound areas were quantified using ImageJ software (NIH). n $\ge 3.$ Statistical significance was assessed by Student's t-test.

### Gelatin degradation (invasion) assay

Fluorescent-coated coverslips were prepared as described previously [21]. Cells were plated on gelatin-coated coverslips in a 24-well plate and fixed after 24 and 48 h later with 4% PFA (v/v) for 15 min at room temperature. Then, filamentous actin and nuclei were stained using Alexa Fluor™ 488 Phalloidin (Invitrogen) or Hoechst 33342 (Invitrogen), respectively. Images were acquired using a confocal microscope (LSM 510; Zeiss) and the degradation areas were quantified using ImageJ software. *n* $\ge 3.$ Statistical significance was assessed by Student's t-test.

### Cell treatment

PDAC cells were seeded in a 6-well plate and after 24 h were treated with 10 ng/ml of recombinant TGF-β1 (rTGF-β1, Peprotech) either alone or in the presence of 80 μM of Vactosertib (Vacto, MedChemExpress) every two days for one week. Cells were harvested with EDTA-trypsin, washed twice with PBS, and collected by centrifugation for RNA extraction. *n* $\ge 3.$ Statistical significance was assessed by Student's t-test.

### Cell growth and chemoresistance assay

Proliferation rates were determined at different time points, as reported in the figures, using the CCK8 assay kit according to the manufacturer’s instruction (Dojindo; Kumamoto, Japan). For the chemoresistance assay, cells were treated with 100 μM of gemcitabine for 48 h. Cell viability was determined using a the CCK-8 assay kit. *n* $\ge 3.$ Statistical significance was assessed by Student's t-test.

### Cell cycle assay

To synchronize the cell cultures, cells were seeded in 6 multi-well plates in growth medium with 10% FBS overnight. Then the cultures were rinsed by PBS and changed to serum free medium. After serum starvation for 24 h, the cells were passaged and released into cell cycle by the addition of serum. For FACS analysis, cell samples were harvested at indicated time points. Cells were trypsinized, washed with PBS, centrifuged, and pellets were fixed in 200 µl of 70% ethanol and stored at -20 °C until use. Cells were centrifuged and pellets resuspended in 200 µl of PBS with 10 µg/mL of RNAse A. Cells were incubated for 1 h at 37 °C prior to resuspension in PI. Cell-cycle analysis was carried out using a CANTO II (BD) flow cytometer. Data were analyzed by FlowJo 9.2 software. *n* $\ge 3.$ Statistical significance was assessed by Student's t-test.

### Apoptosis assay

Attached and floating cells were collected, resuspended and stained with Annexin V (550474; BD Bioscience) after incubation with Annexin V binding buffer (556454, BD PharMingen). Cells were then incubated with PI. Samples were analyzed using a CANTO II (BD) flow cytometer, and data were analyzed using FlowJo 9.2 software. *n* $\ge 3.$ Statistical significance was assessed by Student's t-test.

### Tumor growth

All animal experiments were approved by the local ministry (IACUC protocol #428/2019-PR) and were performed in the animal facility under pathogen-free conditions. Single-cell suspensions of 2.5 × 10^5^ L3.6pl, PANC-1 and #253 (sh empty or sh*LAMC2, EGFP*^+^*or EGFP*^*−*^) cells were subcutaneously injected into 6-week-old nude athymic male mice (Charles River Laboratories). Tumor take was monitored visually and by palpation bi-weekly. Tumor diameter and volume were calculated based on caliper measurements of tumor length and height using the formula: tumor volume = (length x width^2^)/2. Animals were considered to bear a tumor when the maximal tumor diameter was over 2 mm. *n*$\ge 10.$ Statistical significance of tumor volume was assessed by Student's t-test. The overall survival interval of patients was calculated using the Kaplan–Meier method, and differences among subgroups were assessed by the log-rank (Mantel-Cox) test.

### Metastatic assay

Single-cell suspensions of 8 × 10^5^ #253 (EGFP^+^ or EGFP^−^) cells were injected into the tail vein of 6-week-old nude athymic male mice (Charles River Laboratories) (*n* = 5 per group). Vactosertib (40 mg/kg mice) was dissolved in DMSO and administered to mice two times *per* week for three weeks, by intraperitoneal injection. After 2 months from the injection, mice were sacrificed and lungs were resected and fixed in 4% PFA for hematoxylin and eosin staining. n $\ge 7.$ The number of lung metastases were calculated and the statistical significance was assessed by Student's t-test.

### IHC in FFPE

Immunostainings were carried out using 4-μm FFPE tissue sections according to standard procedures. Briefly, after antigen retrieval, samples were blocked with Peroxidase-Blocking Solution (Dako, S202386) for 10 min at room temperature, and samples were then incubated with primary antibodies overnight. Slides were washed with EnVision FLEX Wash Buffer (Dako, K800721), and the corresponding secondary antibody was incubated with the sample for 45 min at room temperature. Samples were developed using 3,3′-diaminobenzidine, counterstained with hematoxylin and mounted. Antibodies against LAMC2 (SIGMA, HPA024638-100U) and GFP (ABCA; ab290) were used at 1:100 dilution overnight at 4 °C in the dark. The nuclei of cell were stained with Hematoxylin. Images were acquired using a digital image scanning (Nanozoomer 2.0HT, Hamamatsu) and cropped using NDP.view2. The area % stain represents the ratio of the summed absolute areas of staining versus the total tissue. The area % stain was analyzed by Fiji ImageJ version v3.2.28. The TMA (Biomax, PA484a) was stained following the above mentioned procedure. Images were acquired using a digital image scanner (Nanozoomer 2.0HT, Hamamatsu) and cropped using NDP.view2. The scoring algorithm takes the proportion of stained cells into consideration, as well as the intensity of the staining. The reactivity was scored in a semi-quantitative manner, which was categorized as low if less than 10% staining was observed in the epithelium; and medium or high based on the intensity if the percentage was between 10–25% and ≥ 25%, respectively. Statistical significance was assessed by Student's t-test.

### Histoscore (H-score)

The intensity of LAMC2 staining was reported based on the H-score method considering the intensity and the % of positive cells. Scoring was as follows: 1 for less than 5%, 2 for 6–15%, 3 for 15–39% and 4 for more than 40%. Statistical significance was assessed by Student's t-test.

### CRISPR/Cas9 knock-in generation in PDAC cells

- **Donor plasmid construction.** 750 bp (LAMC2 construct) of 5’ homology arm (HA) and 3’ homology arm were amplified from PDAC gDNA and cloned in the pDONOR vector. LF2A-EGFP-BGHpA insertion cassette was generated by gene synthesis (Thermo Fisher) and cloned in the 5’HA3’HA previously engineered pDONOR vector.
- **sgRNA design**. Small guide RNAs were designed using the http://crispr.mit.edu web tool. To select for the most suitable sgRNAs, we applied the following criteria: i. localization of the sgRNA as near as possible to the desired site of insertion to maximize homologous recombination efficiency, ii. Cas9-mediated double strand break downstream of the STOP codon to prevent NHEJ-induced indels in the ORF, iii. guide selected to anneal at the intersection between the 5’ homology arm and 3’ homology arm so that the donor plasmid is protected from Cas9 cut, iv. minimum off-target score according to http://crispr.mit.edu and maximum Doench activity score [22]. The sgRNA designed and used to modify PDAC cells was: CCTCAGTTGAGAAATATTTATGG.
- **px330-IRFP Cas9 plasmid construction**. Px330 Cas9 plasmid from Feng Zhang’s laboratory was obtained from addgene (ref. 42230) and was modified by the introduction of a SV40promoter-IRFP expression cassette downstream of Cas9 by FseI – EcoRI. In addition, the BbsI site of IRFP was silenced by site-directed mutagenesis. SgRNAs were cloned in px330-IRFP as described in www.genome-engineering.org/crispr/wpcontent/uploads/2014/05/CRISPR-Reagent-Description-Rev20140509.pdf
- **Nucleofection.** For PDAC cell nucleofection, one million trypsinized PDAC single cells were nucleofected with 3 µg of donor plasmid and 1 µg of px330-IRFP Cas9 corresponding plasmids using the Lonza nucleofector kit V (VVCA-1003) and program A-32 in an Amaxa-II nucleofector following the manufacturer’s protocol.
- **FACS strategy and generation of single cell-derived PDAC cells.** Nucleofected cells were cultured in complete DMEM/RPMI medium for 48 h. Then, IRFP-positive cells were isolated by FACS and cultured in complete medium for 20 days. We sorted the cells for EGFP to confirm donor integration. We then selected the cell population positive and negative for the expression of EGFP (LAMC2-LF2A-EGFP) and we named them EGFP^+^ and EGFP^−^, respectively. Cells were seeded in a 96-well format to derive single-cell clones.
- **Specific genotyping PCRs**. Single-cell derived clones were lysed in buffer consisting of 10 mM Tris, 1 mM EDTA, 1% Tween 20 and 0.4 mg/ml proteinase K for 1 h at 55 °C. The lysate was directly used in the specific integration PCR. For the 5’ specific integration PCR a forward primer upstream of the 5’ homology arm and a reverse primer at the beginning of the inserted cassette were used. Similarly, for the 3’ specific integration PCR a forward primer at the end of the inserted cassette and a reverse primer downstream of the 3’ homology arm were used. The PCR conditions were as follows: DNA Polymerase (BioTools #10012–4103) 95 ºC 2 min × 38 (95 ºC 30 s – 55 ºC 30 s – 72 ºC 1:30 min) 72 ºC 5 min—hold 16 ºC. Sequences of primers used are shown in Table 2.

**Table 2:** Primers used for the specific integration PCR

Locus-insertion		Primer
LAMC2-LF2A-EGFP	5’ specific	F:ACATGCTTGAACAAACGTGATTTTA
		R:CAATTTTCTGTTTGTGTCTGGCTTC
	3’ specific	F:TGAGTAGGTGTCATTCTATTCTGG
		R:TGGTGTTCGAAAAGTAGGATTGAAT

### Bioinformatics analysis

Normalized expression data was downloaded from NCBI GEO (GSE21501, GSE28735, GSE62452 and GSE71729) with the R package GEOquery. GSE21501 consists of 15 tumor samples with resected primary PDAC tumors and 15 patients with metastatic PDAC. GSE28735 consists of 45 tumor samples and matching normal samples. GSE62452 consists of 69 PDAC tumors and 61 normal pancreatic tissue samples from pancreatic cancer patients. GSE71729 consists of 145 primary and 61 metastatic PDAC tumors, 17 cell lines, 46 pancreas and 88 distant site adjacent normal samples A Principal Component Analysis was performed on the entire dataset, which showed a clear separation between tumor and non-tumor samples. Survival was analyzed using the http://gepia2.cancer-pku.cn/#survival. A Median Group cutoff (50% high vs 50% Low) was used. The analysis was performed considering only the classical PAAD subtype (84 patients). The Pearson’s Correlation analysis was performed using the data reported in the http://www.analytics.pancreasexpression.org/index.php?s=icgc. The TCGA dataset is composed by 84 patients with PDAC, the USA cohort is composed by 185 patients with PDAC and the Canadian (CA) cohort is composed by 317 patients with Pancreatic Cancer.

### Gene expression data sets and GSEA analyses

The gene expression data sets used in this study are publicly available. The dataset from Jandaghi et al*.* [23] was downloaded from ArrayExpress (E-MTAB-1791); the data set from Janky et al*.* [24] was downloaded from GEO (GSE62165); the dataset from Bailey et al. was included in a supplementary figure of their published work [25]; the META data set, containing data sets GSE15471, GSE16515, GSE22780, and GSE32688, was generated as described in [26] and the TCGA dataset was downloaded from The Cancer Genome Atlas (TCGA; http://xena.ucsc.edu). A Student's t-test was used to determine significance of expression levels between normal and PDAC samples.

The samples included in the top and bottom quartile of expression of *LAMC2* were compared in GSEA, using the Hallmark gene-set database. The GSEA module of the Genepattern suite from the Broad Institute was used, with 1000 permutations and FDR < 25% was considered statistically significant.

### Single cell data analysis from publicly-available datasets

Human data belong to the TGen database: data (aligned transcripts) were downloaded from GSE154778 [27]. Data from primary tumors was processed with the Seurat package in R, using the FindClusters function with a resolution of 0.5. Graphs were designed with the Seurat package in R as previously described. The ductal signature was clustered according to a 9-genes score identified in Xu et al., [28]. To analyze Moffitt subtypes, cells from each patient were clustered using the FindClusters function in Seurat with a resolution of 0.5. The average expression of 25 marker genes for the ‘basal-like’ or “classical” subtype [29] was then determined for each cluster. Subtype scores for basal-like marker gene expression for each individual cell were calculated using the AddModuleScore function in Seurat [30].

### RNA-sequencing analysis

Total RNA was extracted with Eurogold TRIFAST kit (Euroclone) according to the manufacturer’s instructions. TruSeq stranded mRNA libraries were run on the Illumina NextSeq 500. We generated 40 million reads per lane and paired-end reads from sequencing were trimmed from adaptors and quality checked by the sequencing service. Reads were aligned against the human reference sequence GRCh38 (hg38) from Genome Reference Consortium using BWA (Li 2009). Read counts per gene were determined using the feature Counts method from the Subread R package (Liao 2014) based on the gene information in the gtf available here ftp://ftp.ensembl.org/pub/release-94/gtf/homo_sapiens/Homo_sapiens.GRCh38.94.gtf.gz**.** Gene signatures (Hallmark genesets) were downloaded from GSEA—Molecular Signature Database for Gene set enrichment analysis. RNA-seq data for #253-LAMC2- EGFP high vs. negative cells is available at https://www.ebi.ac.uk/arrayexpress/ under accession number E-MTAB-11597. *n* $\ge 3.$

### Statistical analyses

Results for continuous variables are presented as means ± standard deviation (SD) unless stated otherwise of at least three independent experiments. Treatment groups were compared with the independent samples by Student's t-test. The disease-free interval of patients was calculated using the Kaplan–Meier method, and differences among subgroups were assessed by the log-rank (Mantel-Cox) test. Experiments were performed a minimum of three independent times and always performed with triplicate samples. qPCR were repeated a minimum of five independent times in triplicate. *p* < 0.05 was considered statistically significant. All analyses were performed using GraphPAD Prism7. Correlation analysis were performed applying the Pearson’s correlation coefficient.

## Results

### LAMC2 correlates with poor outcome in PDAC patients

**Fig. 1:**
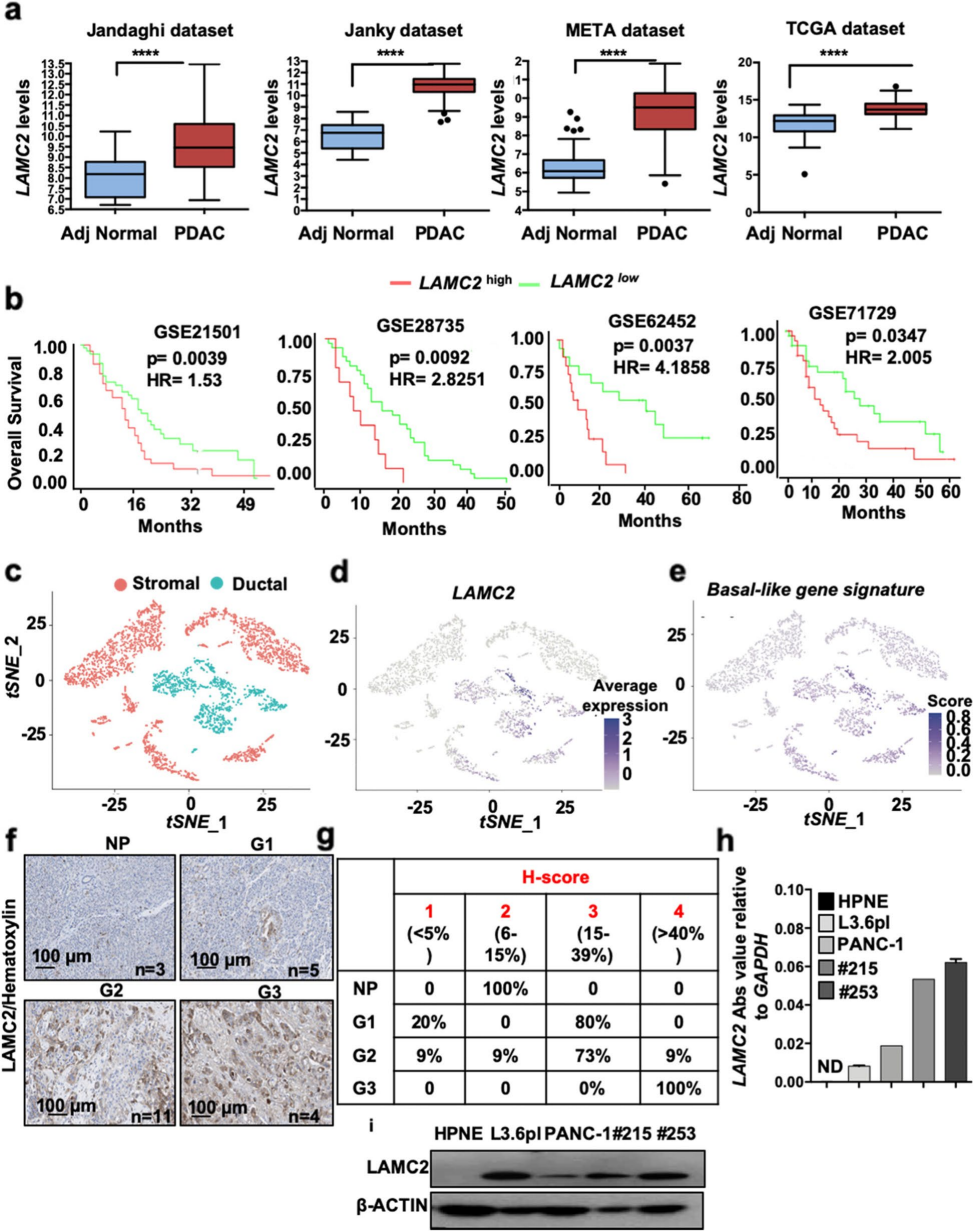
LAMC2 expression in PDAC correlates with poorer outcome. **a** Boxplots illustrating differential expression of *LAMC2* in PDAC tissue versus normal adjacent tissue using the indicated series of transcriptomic data. **** *p* < 0.0001. Statistical significance was assessed by Student's t-test. **b** Kaplan–Meier curves showing overall survival of PDAC patients, stratified according to the median value of *LAMC2* expression. **c** Dimensional reduction plot (DimPlot) of stromal *versus* ductal (tumor) cells identified in PDAC primary tumors by scRNA-Seq. The clusters are color-coded based on cell types identified using known cell type-specific markers and are visualized using t-SNE. **d** Feature Plot for LAMC2 expression in multiple cell types identified in PDAC primary tumors by scRNA-Seq. The clusters are color-coded based on LAMC2 expression and are visualized using t-SNE. **e** Feature Plot for basal-like gene signature expression score in multiple cell types identified in PDAC primary tumors by scRNA-Seq. The clusters are color-coded based on LAMC2 expression and are visualized using t-SNE. **f** Representative images of IHC staining for LAMC2 (brown) in tissue sections from normal pancreas (P) and patients with PDAC tumors at G1, G2 and G3 grade. **g** *H*-score for LAMC2 expression. **h** qPCR analysis for *LAMC2* expression in adherent cells. Data are normalized to GAPDH expression. **i** Western blot analysis of LAMC2 in adherent cells. Parallel β-ACTIN immunoblotting was performed. *n* $\ge 3$

*LAMC2* transcriptional levels were evaluated using the publicly available transcriptome data sets Jandaghi [23], Janky [24], META [26], and TCGA [31]. *LAMC2* mRNA levels were consistently and significantly higher in tumor tissue versus adjacent normal tissue (Fig. 1a). For the GSE21501 [32], GSE28735 [33], GSE62452 [34] and GSE71729 [29] series, well-annotated clinical data are available and showed a significant decrease in median overall survival (OS) for *LAMC2* high-expressing patients compared to *LAMC2* low-expressing patients (Fig. 1b). In the TCGA dataset we observed no differences in OS (*p* value (p) = 0.2289) (Fig. S1f). While in the three GSE (Fig. 1b) we did not observe any association of *LAMC2* expression with sex, alcohol and smoking; in the TCGA dataset we found a significant correlation of increased levels of *LAMC2* with these three parameters, together with age and gender (Fig. S1g).

Using t-distributed stochastic neighbor embedding (t-SNE), we detected 17 segregated cell clusters in the primary PDAC tissues (Fig. S2a) [27]. To categorize the cell identities that those clusters represent, we divided the populations in “ductal” (epithelial cells) and “stromal” (fibroblasts, endothelial cells, and immune cells) (Fig. 1c and Fig. S2b). We observed that the cells present in clusters 3 and 11 were expressing the higher levels (Fig. 1d and Fig. S2c) and percentage (Fig. S2d) of *LAMC2* cells. In both clusters LAMC2 was co-expressed with several poor prognosis markers (e.g., MMP7, KRT19, KRT7 and SOX9) (Fig. S2e) and with the CSC *bona fide* marker PROM1 (i.e., CD133) (Fig. S2f). Interestingly, the LAMC2 cells were also inversely correlated with the “classical” subtype (Fig. S2g) and positively associated with the "basal-like" subtype [29] (Fig. 1e and Fig. S2h), which is usually linked with poorly differentiated and extremely aggressive tumors.

Next, LAMC2 immunohistochemistry (IHC) was performed using a tissue microarray (TMA) composed of 18 PDAC samples and three NP (Normal Pancreas) tissues (Fig. 1f and Fig. S1a). LAMC2 expression was classified as 1 to 4 based on the *H*-score and stratified for PDAC stage 1 to 3, and the results revealed significantly increased LAMC2 levels in PDAC patients versus NP, and a clear correlation with disease progression (Fig. 1g and Fig. S1b).

We also queried the Human Protein Atlas database for LAMC2 [35]. A total of 12 patients with PDAC were classified based on LAMC2 staining, intensity and quantity. We observed that 9 out of 12 samples showed strong LAMC2 staining compared to 3 samples with medium levels of LAMC2; eleven samples presented strong (cytoplasmic/membranous) intensity for LAMC2 whereas one sample was classified as LAMC2 moderate; the distribution of stained cells showed one sample with < 25%, two samples with 25–75% and nine sample with > 75% (Fig. S1c). In line with the observed significant association of LAMC2 expression with age and gender in the TMA, a t-test analysis of LAMC2 staining in the Human Protein Atlas samples revealed significant difference in LAMC2 H-score > 30% in male respect to female patients with PDAC (*p* < 0.05). There was also increased LAMC2 (H-score > 30%) in the group of patients aged > 60 years compared to patients aged < 60 (*p* < 0.05) (Fig. S1d-e). In vitro, we confirmed the absence of LAMC2 expression (gene and protein) in Normal Pancreatic cancer cells (i.e., HPNE [36] compared to two established pancreatic cancer cell lines (L3.6pl and PANC-1) and two human PDAC-derived primary cultures (#215 and #253) [4, 37] (Fig. 1h and Fig. 1i). Taken together, the sum of these results suggest a strong clinical relevance for LAMC2 in PDAC.

### LAMC2 expression correlates with stemness

**Fig. 2:**
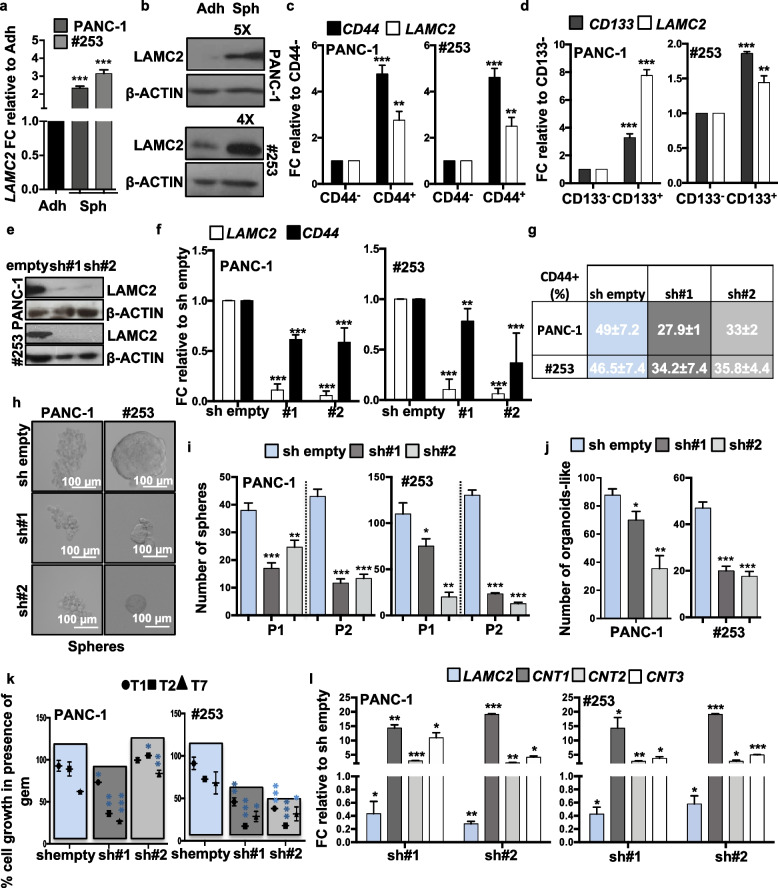
LAMC2 expression correlates with stemness and chemoresistance. **a** qPCR analysis for *LAMC2* gene expression in adherent cells versus spheres. Data are normalized to *GAPDH* and are presented as fold change in gene expression relative to adherent cells. **b** Western blot analysis for LAMC2 in adherent cells versus spheres. Parallel β-ACTIN immunoblotting was performed. **c** qPCR analysis for *CD44* and *LAMC2* gene expression in CD44^+^ sorted cells. Data are normalized to *GAPDH* and are presented as fold change in gene expression relative to CD44^−^ cells. **d** qPCR analysis for *CD133* and *LAMC2* gene expression in CD133^+^ sorted cells. Data are normalized to *GAPDH* and are presented as fold change in gene expression relative to CD133^−^ cells. **e** Western blot analysis for LAMC2 in sh empty and *LAMC2* knockdown cells. Parallel β-ACTIN immunoblotting was performed. **f** qPCR analysis for *LAMC2* and *CD44* expression for sh empty *versus LAMC2* knockdown cells. Data are normalized to *GAPDH* expression and are presented as fold change in gene expression relative to sh empty. **g** Flow cytometry quantification of CD44 in sh empty and *LAMC2* knockdown cells. **h** Representative images of sh empty and *LAMC2* knockdown cells grown as spheres. **i** Sphere formation capacity of sh empty and *LAMC2* knockdown cells. P1 = 1^st^ generation; P2 = 2^nd^ generation. **j** Organoid formation capacity for sh empty *versus LAMC2* knockdown cells. **k** Growth capacity of sh empty and *LAMC2* knockdown cells in the presence of 100 μM Gemcitabine (GEM). **l** qPCR analysis for *LAMC2, CNT1, CNT2* and *CNT3* genes in sh empty *versus LAMC2* knockdown cells. Data are normalized to *GAPDH* expression and are presented as fold change in gene expression relative to sh empty. *p < 0.05, **p < 0.005, ***p < 0.0005. *n* $\ge 3.$ Statistical significance was assessed by Student's t-test

As poor outcome for PDAC has been related to the TIC/CSC content [38–40], we hypothesized that higher expression of LAMC2 may be associated with stemness. First, we correlated the levels of LAMC2 (mRNA and protein) for cells cultured in adherent (Adh; enriched for differentiated cells) *versus* anchorage-independent conditions (Spheres, Sph; enriched for CSCs) [4]. Quantitative real-time PCR (qPCR) (Fig. 2a and Fig. S3a) and western blotting (Fig. 2b and Fig. S3b) confirmed that LAMC2 was significantly upregulated in spheres compared to adherent cells. Concomitantly, the stemness-associated genes (i.e., *CD44* and *CD133*) were also overexpressed in spheres compared to adherent cells (Fig. S3c). To corroborate our findings we also observed a direct correlation between *CD44* and *LAMC2* expression in the TCGA dataset by performing a Pearson’s Correlation analysis (Fig. S3d). We recently demonstrated that the reduced expression of L1CAM represents a hallmark of stemness [15]. Consistently, spheres also showed reduced *L1CAM* expression (Fig. S3c). Since LAMC2 is an adhesion molecule, we asked whether the increased expression of LAMC2 in spheres was related to differences in culture conditions (presence/absence of serum and monolayer/suspension). First, we compared LAMC2 expression in adherent cells versus sphere-derived cells by exchanging the medias. Specifically, we grew cells as monolayers or in suspension using either medium containing 10% FBS (Diff. medium) or CSC medium without FBS. We found that LAMC2 upregulation was not related to the medium but rather to culture conditions (Fig. S3d) and correlated with the enrichment for CSCs (characterized by the upregulation of CD44 and CD133 and the downregulation of L1CAM) in spheres. No differences were observed in sphere morphology, size or number after short (24 h) or long (7 days) time in Diff. medium compared to CSC medium (Fig. S3f). Moreover, we tested the differentiation potential of the spheres as an important feature of cancer cell plasticity. For this purpose, we cultured L3.6pl, PANC-1 and #253 cells as spheres in the CSC medium for 7 days, and then plated them as monolayers in Diff. medium for 4 days (i.e., differentiated spheres). By qPCR we found that the expression of *LAMC2* was increased in spheres compared to the parental adherent cells and the levels were reduced in differentiated spheres (Fig. S3g). At the same time, the expression of stemness genes (e.g., *ABCG2*, *CD133,* and *SOX2*) was significantly higher in spheres, compared to adherent cells, and the levels decreased in the differentiated spheres. As expected, *L1CAM* levels were lower in the spheres than in adherent cultures, and its expression was restored in spheres upon differentiation (Fig. S3g). Interestingly, FACS-sorted CD44^high^ or CD133^high^ cells, showed a significant increase in *LAMC2* compared to their respective low/negative counterparts (Fig. 2c-d and Fig. S3h-i). Lastly, *in vitro* treatment of L3.6pl, PANC-1, #215 and #253 with 100 μM of gemcitabine (Gem) revealed a significant increase in *LAMC2* levels in Gem-treated cells compared with vehicle treated cells (Ctrl) (Fig. S3j). Taken together these data demonstrate that LAMC2 is enriched in the “standard” CSC population(s).

### Loss of LAMC2 affects stemness

To further validate the above findings we next silenced *LAMC2* in L3.6pl, PANC-1, #215 and #253 using two different lentiviral shRNA constructs (sh#1 and sh#2) (Fig. 2e-f and Fig. S4a-b). Upon knockdown of *LAMC2*, no apparent morphological changes were noted for cells cultured as monolayers (Fig. S4c). While no changes in cell cycle were recorded, we observed modest alternations in cell viability (Fig. S4d-e); however, reduced expression of *LAMC2* did not translate into enhanced apoptosis (Fig. S5a). By qPCR we observed that sh*LAMC2* cells exhibited significantly decreased mRNA levels for *CD44* compared with mock-infected cells (sh empty) (Fig. 2f), which was confirmed by flow cytometry (Fig. 2g and Fig. S5b). Only minor (not significant) changes were observed for both *CD133* mRNA (qPCR; Fig. S5c) and protein expression (flow cytometry, Fig. S5d). Consistently, we found that the number and size of both spheres (Fig. 2h-i and Fig. S5e-g) and organoid-like structures (Fig. 2j and Fig. S5h) were significantly decreased in sh*LAMC2* cells compared to sh empty cells.

**Fig. 3:**
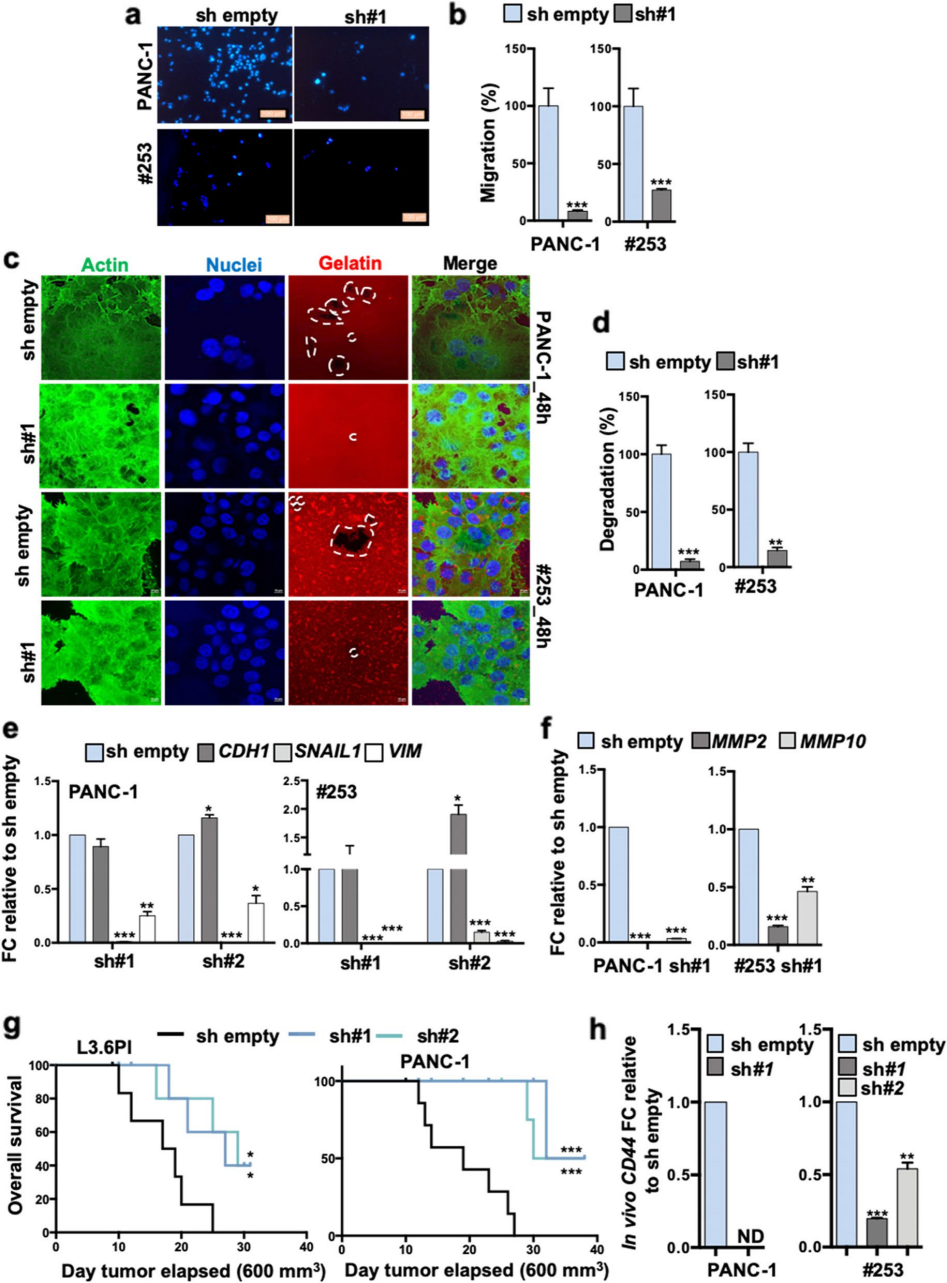
Loss of *LAMC2* reduces tumorigenicity. **a** Migration assay for sh empty *versus LAMC2* knockdown cells. The nuclei were stained with DAPI (blue). **b** Migratory potential of sh empty *versus LAMC2* knockdown cells. **c** Representative images of gelatin degradation for sh empty *versus LAMC2* knockdown cells. Nuclei were stained with Hoechst 33342 (blue), green represents actin (Alexa Fluor™ 488 Phalloidin) and red illustrates gelatin (Rodhamine). The white dashed lines circles indicates the areas of degradation. **d** Invasive potential of sh empty *versus LAMC2* knockdown cells. **e** qPCR analysis for EMT genes in sh empty and *LAMC2* knockdown cells. Data are normalized to *GAPDH* and are presented as fold change in gene expression relative to sh empty. **f** qPCR analysis for *MMP2* and *MMP10* gene expression in sh empty and *LAMC2* knockdown cells. Data are normalized to *GAPDH* and are presented as fold change in gene expression relative to sh empty. **g** Kaplan–Meier curve for sh empty and *LAMC2* knockdown cells subcutaneously xenografted into nude athymic mice. *n* ≥ 10. **h** qPCR analysis for *CD44* gene expression in sh empty and *LAMC2* knockdown cells isolated from respective tumors. Data are normalized to *GAPDH* and are presented as fold change in gene expression relative to sh empty. *p < 0.05, **p < 0.005, ***p < 0.0005. *n* $\ge 3.$ Statistical significance was assessed by Student's t-test. For the Kaplan–Meier curve, the statistical significance was assessed by the log-rank (Mantel-Cox) test

Next, we tested knockdown cells for chemoresistance and found that sh*LAMC2* cells were more sensitive to gemcitabine compared to sh empty cells (Fig. 2k). Gemcitabine is transported by multiple active nucleoside transporters (e.g. *CNT1, CNT2* and *CNT3*) and we found augmented expression for all of them in sh*LAMC2* cells compared to sh empty cells (Fig. 2i). Transmigration (Fig. 3a-b and Fig. S5i-j) and wound healing (Fig. S5k) assays confirmed that sh*LAMC2* cells were less aggressive/migratory compared to sh empty cells. In addition, in a gelatin degradation (Fig. 3c-d and Fig. S6a-b) assay we observed reduced invasive potential of sh*LAMC2* cells compared with their sh empty counterpart, as confirmed by the reduced presence of degradation areas (white circles), attributable to the lower aggressiveness of knockdown cells. Of note, as shown above (Fig. S4d), we did not observe decreased proliferation in sh*LAMC2* cells compared with sh empty cell, thereby excluding that the smaller number of transmigrated sh*LAMC2* cells was merely due to their reduced proliferation. Tumor cell invasion requires loss of cell–cell interactions, and is often associated with the epithelial–to-mesenchymal transition (EMT). qPCR analysis revealed upregulation of the epithelial marker *CDH1* (E-Cadherin), whereas the mesenchymal transcription factors (i.e. *SNAIL1* and *VIMENTIN*) were downregulated in sh*LAMC2* cells compared with sh empty cells (Fig. 3e and Fig. S6c-d). Increased matrix metalloproteases (MMPs) levels are, nowadays, considered an important sign of the pro-tumorigenic and invasive potential of tumor cells. In line with the aforementioned observations, we observed a significant reduced expression of *MMP2* and *MMP10* in sh*LAMC2* cells compared with sh empty cells (Fig. 3f and Fig. S6e). Lastly, we subcutaneously injected 250,000 L3.6pl, PANC-1, #215 or #253 sh empty or sh*LAMC2* cells into nude mice and observed that the tumors generated by sh*LAMC2* cells were: fewer, smaller and showed delayed initiation compared with those generated by sh empty cells (Fig. 3g and Fig. S6f-g). Importantly, qPCR analysis confirmed that *LAMC2* was still downregulated in the excised sh*LAMC2* tumors, and that they exhibited reduced levels of mesenchymal and metalloprotease genes (Fig. S6h) as well as non-detectable (for PANC-1) or significantly reduced levels of *CD44* cell surface expression (Fig. 3h).

### Generation of *LAMC2*-EGFP knock-in human pancreatic cancer cells

**Fig. 4:**
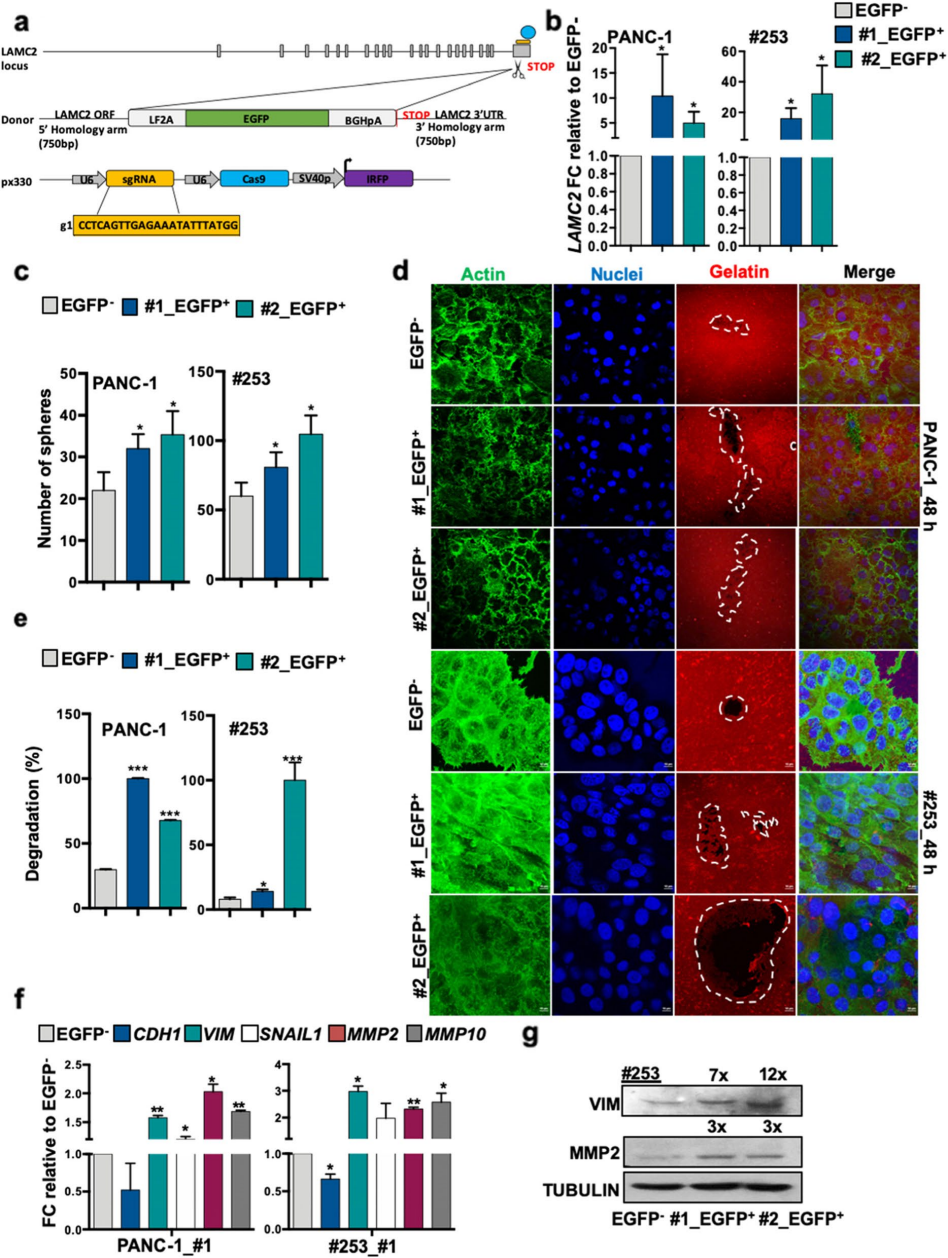
Generation of *LAMC2*-EGFP knock-in human pancreatic cancer cells. **a** Design of LAMC2‐EGFP donor and CRISPR/Cas9 sgRNA vectors. Blue circle represents the CRISPR/Cas9 protein complex and the yellow box underneath illustrates the guide RNA. **b** qPCR analysis for *LAMC2* gene expression in EGFP^+^ and EGFP^−^ cells. Data are normalized to *GAPDH* and are presented as fold change in gene expression relative to the EGFP^−^ counterpart. **c** Sphere formation capacity for EGFP^+^ *versus* EGFP^−^ cells. **d** Representative images of gelatin degradation for EGFP^+^ *versus* EGFP^−^ cells. Nuclei were stained with Hoechst 33342 (blue), green represents actin (Alexa Fluor™ 488 Phalloidin) and red illustrates gelatin (Rodhamine). The white dashed line circles indicates the areas of degradation. **e** Invasive potential of sh empty *versus LAMC2* knockdown cells. **f** qPCR analysis for EMT and *MMP2* and *MMP10* gene expression for EGFP^+^ *versus* EGFP^−^ cells. Data are normalized to *GAPDH* and are presented as fold change in gene expression relative to the EGFP^−^ cells. **g** Western blot analysis of VIM and MMP2 in EGFP^+^ and EGFP^−^ cells. Parallel Tubulin immunoblotting was performed. *p < 0.05, **p < 0.005, ***p < 0.0005. *n* $\ge 3.$ Statistical significance was assessed by Student's t-test

LAMC2 is a secreted molecule present in the extracellular matrix, which rendered sorting of the cells based on LAMC2 expression and their subsequent downstream analysis difficult. We thus designed a strategy using CRISPR/Cas9-mediated homologous recombination to mark *LAMC2* cells. For these experiments, we selected L3.6pl, PANC-1 and #253 cells. The targeting strategy is summarized in Fig. 4a and detailed in the Materials and Methods section. In brief, we designed a Cas9 single-guide RNA complementary to sequences overlapping the stop codon of the *LAMC2* locus and generated a donor vector that contained *LAMC2* homology arms flanking an EGFP reporter cassette positioned immediately upstream of the stop codon. We added an LF2A self-cleavage peptide [41] in frame with EGFP so that the *LAMC2*-EGFP locus would be transcribed as a single mRNA, whereas the resulting polypeptide would be cleaved in the two encoded proteins, LAMC2 and EGFP (Fig. 4a). Next, we nucleofected L3.6pl, PANC-1 and #253 cells with the donor vector together with a guide-RNA-Cas9 (guide), and after 48 h we sorted cells that had incorporated the sgRNA-Cas9 px330 vector, which had an incorporated IRFP selectable cassette [42] (IRFP^+^ cells) (Fig. S7a). Approximately 26–31% in L3.6pl, 33% in PANC-1 and 4–6% in #253 IRFP^+^ cells expressed EGFP after 20 days in culture (Fig. S7b). Subsequently, we generated single cell-derived cultures and assessed the specific integration of the EGFP reporter cassette by PCR (Fig. S7c). These analyses showed that 100% of L3.6pl, 100% of PANC-1 and 37.5% (3 out of 8) of #253 clones had correctly integrated the EGFP reporter in the *LAMC2* locus. In these single cell-derived knock-in cell cultures, every culture was composed of an admixture of cells expressing distinct EGFP levels (Fig. S7d). *LAMC2*-EGFP^+^ cells isolated by FACS expressed the highest *LAMC2* mRNA levels confirming that EGFP correctly reported endogenous LAMC2 expression (Fig. 4b and Fig. S7e).

### Characterization of human *LAMC2*-EGFP PDAC cells *in vitro* and *in vivo*

We next investigated the properties of the LAMC2-EGFP^+^ cells. Morphologically we did not observe, as expected, significant changes in the LAMC2-EGFP-clones (EGFP^+^#1 and EGFP^+^#2) compared to control cells (EGFP^−^) (Fig. S7f). Likewise, the cell cycle status was essentially unaltered between LAMC2-EGFP-clones and EGFP^−^ cells (Fig. S7g). To confirm that the LAMC2-EGFP^+^-clones maintained their intrinsic CSC properties, we examined their ability to grow as spheroids in standard sphere-forming medium. After 7 days of culture, the numbers of spheres formed were greater among LAMC2-EGFP^+^-clones versus the corresponding EGFP^−^ cells (Fig. 4c and Fig. S7h). Moreover, the size (but not the number) of organoid-like structures was significantly increased in LAMC2-EGFP^+^-clones compared with EGFP^−^ cells (Fig. S8a-c). A gelatin degradation assay also confirmed that LAMC2-EGFP^+^-clones were more aggressive compared with EGFP^−^ cells (Fig. 4d-e). qPCR analysis showed that LAMC2-EGFP^+^-cells indeed exhibited increased expression levels for mesenchymal genes (*VIM* and *SNAIL1*) and metalloproteases genes (*MMP2* and *MMP10*), respectively, compared to levels for the corresponding EGFP^−^ cells (Fig. 4f-g and Fig. S8d).

**Fig. 5:**
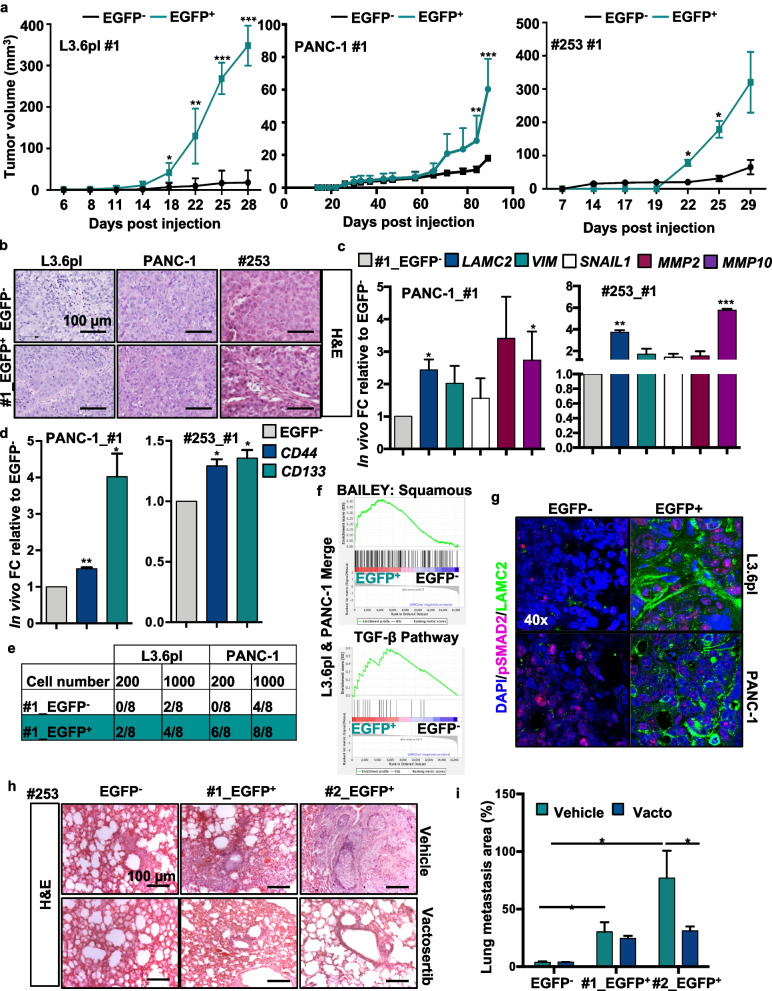
Pharmacological inhibition of TGF-β signaling blocks LAMC2-induced metastasis. **a** Volume of tumors formed following subcutaneously injection of EGFP^+^ and EGFP^−^ cells in nude athymic mice. *n* ≥ 10. **b** Representative H&E-stained sections of xenografts derived from EGFP^+^ or EGFP^−^ cells. **c** qPCR analysis for *LAMC2,* EMT*, MMP2* and *MMP10* gene expression in EGFP^+^ or EGFP^−^ cells, isolated from tumors. Data are normalized to *GAPDH* and are presented as fold change relative to EGFP^−^. **d** qPCR analysis or *CD44* and *CD133* expression in EGFP^+^ or EGFP^−^ cells isolated from tumors. Data are normalized to *GAPDH* and are presented as fold change in gene expression relative to EGFP^−^. **e** Number of tumors generated by subcutaneous injection of EGFP^+^ or EGFP^−^ cells. **f** Enrichment plot for EGFP^+^ *versus* EGFP^−^ cells isolated by FACS from subcutaneous tumors. **g** Representative immunofluorescence images for pSMAD2 (violet), LAMC2 (green) and nuclei (blue, DAPI) in tumor sections derived from EGFP^−^ or EGFP^+^ cells subcutaneously xenografted in nude athymic mice. **h** qPCR analysis for *LAMC2* expression in PDAC cells treated with 10 ng/ml of rTGF-β1 in the presence or absence of 80 μM Vactosertib. Data are normalized to *GAPDH* and are presented as fold change in gene expression relative to control. **i** Representative H&E-stained sections of lungs following tail vein injection of EGFP^+^ or EGFP^−^ tumor cells. Mice were treated with Vactosertib (40 mg/kg mice) or vehicle. *p < 0.05, **p < 0.005, ***p < 0.0005. *n* $\ge 5.$ Statistical significance was assessed by Student's t-test

Next, we subcutaneously inoculated nude athymic mice with LAMC2-EGFP^+^ or EGFP^−^ cells and evaluated their *in vivo* tumorigenicity. LAMC2-EGFP^+^-cells were able to generate earlier and bigger tumors compared to EGFP^−^ cells (Fig. 5a and Fig. S8e-f). Histological analyses revealed that the tumor xenografts derived from LAMC2-EGFP^+^-clones displayed a glandular organization and increased desmoplasia compared with tumor xenografts derived from EGFP^−^ cells (Fig. 5b and Fig. S8g). A substantial proportion of the epithelial compartment of the tumor showed LAMC2-EGFP^+^ cells, with their EGFP levels varying considerably (Fig. S8g-h). Tumors generated from LAMC2-EGFP^+^ cells were populated with LAMC2-EGFP^+^ and EGFP^−^ tumor cells thus implying that LAMC2-expressing cells undergo self-renewal and differentiation during tumor formation and expansion (Fig. S8g-h). Of note, xenografts generated by EGFP^−^ cells contained both LAMC2-EGFP^+^ and EGFP^−^ cells (Fig. S8g), indicating plasticity. By qPCR analysis we determined that tumors derived from LAMC2-EGFP^+^ cells expressed more than 5-fold higher levels of *LAMC2* than tumors derived from EGFP^−^ cells (Fig. 5c and Fig. S8i). Moreover, LAMC2-EGFP^+^-derived tumors were enriched for *VIM*, *SNAIL1*, *MMP2* and *MMP10* (Fig. 5c and Fig. S8i) and downregulated *CDH1* (Fig. S8j). Interestingly, EGFP^+^ cells displayed enhanced expression of CSC markers *CD44* and *CD133* compared to EGFP^−^ cells (Fig. 5d). The expression levels for the aforementioned genes were comparable to levels observed *in vitro*. To assess the capacity of these tumor cell populations to serially propagate the disease into secondary hosts, we inoculated 200 or 1,000 LAMC2-EGFP^+^ or EGFP^−^ epithelial tumor cells into new recipient mice. These experiments showed that the LAMC2-EGFP^+^ cell population was strongly enriched for TIC cells compared to their differentiated EGFP^−^ counterparts (Fig. 5e). Specifically, the LAMC2-EGFP^+^ fraction from L3.6pl cells formed tumors with 200 cells (2/8) while the EGFP^−^ fraction formed only very small tumors with 1,000 cells (4/8). For the PANC-1 cell line, 200 isolated LAMC2-EGFP^+^ cells initiated tumors (6/8) whereas 1,000 cells were required to initiated tumors in the EGFP^−^ (4/8) group (Fig. 5e).

### Inhibition of transforming growth factor beta (TGF-β) signaling blocks LAMC2-induced metastasis

To identify key cellular pathways defining the specific features of LAMC2 expressing cells, a transcriptomic analysis was performed from RNA-seq of LAMC2-EGFP^+^ and EGFP^−^-derived tumors (Fig. 5f and Fig. S9). Gene set enrichment analysis (GSEA) revealed a pro-metastatic signatures in LAMC2-EGFP^+^-derived tumors. Interestingly, we observed that the LAMC2-EGFP^+^-derived tumors showed significant enrichment in the squamous subtype transcriptional profile (Fig. 5f), which has been linked to hypermethylation and down-regulation of genes determining endodermal identity in the pancreas, and is associated with poor outcome [25]. Moreover, we found enrichment for transcripts related to the TGF-β (Fig. 5f), focal adhesion, hypoxia, and MAP Kinases pathways (Fig. S8 and S10a). Encouraged by the above results we examined the link between LAMC2 expression and TGF-β/SMAD signaling. By Pearson’s correlation analysis we confirmed a link between LAMC2 and TGF-β signaling-associated genes (i.e., *TGFβR1*, *TGFβR2*, *TGF-β1*, *TGF-β2*, *SMAD2* and *SMAD4*) (Fig. S10b). We then treated PDAC cells with recombinant TGF-β1 or TGF-β2 and observed a strong increase of *LAMC2* expression (Fig. S10c-d), which was significantly reduced by the co-treatment with Vactosertib, a new clinical-grade inhibitor of TGF-β receptor I (Fig. S10d). By western blotting analysis for SMAD2 phosphorylation (pSMAD2) we did not observe any differences in LAMC2-EGFP^+^ compared to LAMC2-EGFP^−^ cells both in the whole cell lysate (upper panel) or in the cellular fractionation (lower panel) (Fig. S10e). In contrast, by flow cytometry we observed a significant increase in the TGFβ receptor 1 (TGFβR1-ALK5) expression in LAMC2-EGFP^+^ cells compared to their EGFP^−^ counterparts, pointing out a potential capacity of these cells to respond more efficiently to TGF-β stimuli (Fig. S10f). We therefore tested this hypothesis by treating both LAMC2-EGFP^+^ and LAMC2-EGFP^−^ cells with rTGF-β1 and by flow cytometry (Fig. S10g) observed a significant increase of the double positive population for EGFP (LAMC2 reporter) and CD44 in LAMC2-EGFP^+^, strengthening also the correlation between LAMC2 and CD44. Interestingly, by immunofluorescence analysis we observed an increase in LAMC2 and nuclear pSMAD2 expression in LAMC2-EGFP^+^-derived tumors respective to EGFP^−^ cells (Fig. 5g).

Metastatic PDAC is caused by disseminated cancer cells that maintain the capacity to initiate new lesions in distant organs [43], which for PDAC are mainly the liver and lung. To assess the ability of the LAMC2-EGFP^+^ cells to generate metastases, we inoculated them as dissociated cells via the tail vein in nude athymic mice. While hardly any metastases were formed following injection of thousands of EGFP^−^ cells, EGFP^+^ cells were highly metastatic (Fig. 5h-i). Importantly, treatment of mice with Vactosertib completely abrogated the capacity of LAMC2-EGFP^+^ cells to form metastases (Fig. 5h-i), underlying that the TGF-β/SMAD signaling pathway is a key driver of LAMC2^+^ CSC-mediated metastases *in vivo*.

## Discussion

TIC or CSC have been proposed to represent key drivers for tumor progression and metastasis formation in PDAC. However, to date, there are still no effective and translatable therapeutic strategies that actually eradicate this subset of highly aggressive cancer cells. Efficiently targeting TICs appears to be essential to achieve a cure, due to their self-renewal capacity, resistance to conventional chemotherapies [44], and role in tumor relapse. Despite significant advances in our understanding of TIC biology, the identification of specific markers to help isolate these cells remains largely debated, incompletely established, and limited to a handful of markers that are neither solely expressed on TICs nor expressed across all TIC subpopulations. Here, we demonstrated that PDAC cells with high expression of LAMC2 were highly metastatic, consistent with the inverse correlation between LAMC2 expression and patient survival.

LAMC2 levels have already been associated with poor prognosis in several human cancers due to its effects on cancer cell proliferation, migration and invasion. In pancreatic cancer, by performing both integrated bioinformatics analyses and proteomic assays, its potential as a new putative biomarker has emerged [45]. Of note, elevated serum levels of LAMC2 in patients with pancreatic cancer make it an attractive serum-based diagnostic marker [46]. Nevertheless, no evidences regarding LAMC2 in driving the stem properties and the metastatic capacities of PDAC cells have been reported.

Here, we showed that the knockdown of *LAMC2* resulted in decreased self-renewal, migration, invasion, tumorigenicity and chemoresistance and *in vivo* knockdown of *LAMC2* reduced tumorigenic potential, thus suggesting that silencing of LAMC2 counteracts the TIC phenotype and consequently PDAC aggressiveness. The CRISPR/Cas9 technology employed in this study allowed us to study human tumors through genetic manipulations that had only been feasible to date in animal models. This technological advance is particularly well suited to analyze phenotypic diversity of cell populations within cancers as it enables genetic editing of genes. For example, the labelling of distinct tumor cells with specific marker genes, which are not necessarily expressed at the cell surface such as LAMC2. Therefore, tumors generated from edited cells reflect the behavior of a single TIC lineage in a genetically homogenous mutational background. For the first time, this approach now enabled us to explore the behavior of LAMC2-EGFP^+^ cells in intact tumors, and helped us to demonstrate directly how this cell population contributes to PDAC tumor cell growth and dissemination.

First, we showed that LAMC2-EGFP^+^ cells displayed EMT molecular traits concomitant with an increased migratory activity, which explained the enhanced metastatic features of these cells when injected in immunocompromised mice. Transcriptomic analyses of LAMC2-EGFP^+^ cell-derived tumors revealed a signature consistent with the squamous subtype as defined in the Bailey classification [25]. In this classification, squamous is the molecular subtype of PDAC with the worse prognosis. In addition, enrichment of gene networks involved in inflammation, hypoxia, metabolic reprogramming, TGF-β signaling, MYC pathway activation and its target genes were also observed and are key features of squamous PDAC tumors [47].

Interestingly, the activation of TGF-β signaling plays a key role in promoting TIC migration and metastasis [4, 15, 48, 49]. Cytokines released in the tumor microenvironment significantly contribute to maintain the undifferentiated state and clonogenic activity of TICs [50]. Specifically, here we found TGF-β1 to be a potent inducer of LAMC2 expression. *In vivo,* a similar effect might be induced by TGF-β1 derived from stromal and/or LAMC2-high cells.

While stromal cells seem to play a key role in the production of TGF-β1 [51], it is likely that autocrine production of TGF-β1 may contribute to promoting the metastatic activity of LAMC2-EGFP^+^ cells. Intriguingly, Vactosertib completely prevented metastasis formation by LAMC2-EGFP^+^ cells. Vactosertib is a novel and orally administered transforming growth factor-β (TGF-β) type I receptor inhibitor that is currently in clinical testing for other cancer types (https://www.clinicaltrials.gov/ct2/show/NCT04258072). Therefore, our findings may have important therapeutic implications for PDAC. Although some LAMC2-low cells also bear modest tumorigenic potential and are capable of giving rise to small tumors, the vast majority of PDAC tumor cell tumorigenic capacity seems to be confined to the LAMC2-high population, which contains virtually all the cells with high level of TGF-β activation. Because tumorigenic capacity is a prerequisite to successfully seed metastatic lesions, it is likely that the relative density of TICs in PDAC tumor directly correlates with the metastatic propensity of tumor lesions. In this context, LAMC2 could serve as a key functional biomarker.

## Conclusion

Our study identified a tumor-initiating cell population in pancreatic cancer characterized by high levels of LAMC2, which undergoes self-renew and differentiation toward a squamous-like phenotype. Targeting of LAMC2-expressing cells with the novel transforming growth factor beta (TGF-β) signaling inhibitor Vactosertib, could be used to suppress CSC-related metastasis in PDAC patients.

## Supplementary Information

**Additional file 1: Figure S1.** Increased LAMC2 expression is associated with an unfavorable outcome in PDAC. (a) Representative images of IHC staining for LAMC2 (brown) in tissue sections from pancreatic islets. (b) *H*-score for LAMC2 expression. (c) Number of PDAC tumors (Human Protein Atlas database) classified based on LAMC2 IHC staining, intensity and quantity. (d) Patients distribution according to gender, age and TNM. (e) *H-score* and grade distribution according to gender and age. (f) Kaplan-Meier curves showing overall survival of PDAC patients, stratified according to the median value of *LAMC2* expression based on the TCGA dataset. (g) Kaplan-Meier curves showing overall survival of PDAC patients, stratified according to the median value of *LAMC2* expression for gender, alcohol consumption and smoking based on the TCGA dataset. *p<0.05. Statistical significance was assessed by Student's t-test.

**Additional file 2: Figure S2.** Increased LAMC2 expression is associated with aggressive signature. (a) Dimensional reduction plot (DimPlot) of multiple cell types identified in PDAC primary tumors by single-cell RNA sequencing (scRNA-Seq). The clusters are color-coded based on cell types identified using known cell type-specific markers and are visualized using t-Distributed Stochastic Neighbor embedding (t-SNE). (b) Violin plot showing expression levels of LAMC2 in stromal *versus *ductal (tumor) cells identified in PDAC primary tumors by scRNA-Seq. (c) Violin plot showing the expression of LAMC2 in the 17 clusters identified in PDAC primary tumors by scRNA-Seq. (d) Percentage of LAMC2 positive cells in the different clusters identified in PDAC primary tumors by scRNA-Seq. (e) DotPlot depicting expression of ductal genes in the 17 clusters identified in PDAC primary tumors by scRNA-Seq. The size of the dots represents the percentage of cells expressing the gene within a cluster, whereas the colour intensity represents the average expression level. (f) DotPlot depicting expression of stem markers genes in the 17 clusters identified in PDAC primary tumors by scRNA-Seq. The size of the dots represents the proportion of cells expressing the gene whereas the colour intensity represents the average expression level. (g) Scatter plot of LAMC2 normalized expression in the Moffitt’s classical gene signature score of PDAC. (h) Scatter plot of LAMC2 normalized expression in the Moffitt’s basal-like gene signature score of PDAC.

**Additional file 3: Figure S3.** LAMC2 expression correlates with CSC content and function. (a) qPCR analysis of *LAMC2 *gene expression in adherent cells versus spheres. Data are normalized to *GAPDH* and are presented as fold change in gene expression relative to adherent cells (indicated as Adh). (b) Western blot analysis of LAMC2 in adherent cells versus spheres. Parallel β-ACTIN immunoblotting was performed. (c) qPCR analysis for *CD44, CD133* and *L1CAM* genes in adherent cells versus spheres. Data are normalized to *GAPDH* and are presented as fold change in gene expression relative to adherent cells. (d) Correlation between *CD44* and *LAMC*2 in PDAC samples from the TCGA dataset. The p value is based on Pearson’s Correlation. (e) qPCR analysis for* LAMC2 *in PDAC cells grown in different culture conditions. (f) qPCR analysis for* LAMC2, ABCG2*, *CD133*, *L1CAM* and *SOX2 *in Adh *vs *Spheres *vs* Differentiated cells. Data are normalized to *GAPDH* and are presented as fold change in gene expression relative to adherent cells. (g) Representative images of L3.6pl and PANC-1 cells growth after short (24 hours) or long (7 days) times in Diff. medium compared to CSC medium. (h) qPCR analysis for *CD44* and *LAMC2* genes in CD44^+^ sorted cells. Data are normalized to *GAPDH* and are presented as fold change in gene expression relative to CD44^-^cells. (i) qPCR analysis for *CD133* and *LAMC2* genes in CD133^+^ sorted cells. Data are normalized to *GAPDH* and are presented as fold change in gene expression relative to CD133^-^ cells. (j) qPCR analysis for *LAMC2* in PDAC cells treated with 100 µM of gemcitabine. Data are normalized to *GAPDH* and are presented as fold change in gene expression relative to control cells (untreated). *p<0.05, **p<0.005, ***p<0.0005. *n*$\ge 3.$ Statistical significance was assessed by Student's t-test.

**Additional file 4**: **Figure S4.** Knockdown of *LAMC2* does not affect cell growth. (a) Western blot analysis of LAMC2 in sh empty and *LAMC2* knockdown cells. Parallel β-ACTIN immunoblotting was performed. (b) qPCR analysis for *LAMC2* and *CD44* gene expression in sh empty and *LAMC2* knockdown cells. Data are normalized to *GAPDH* and are presented as fold change in gene expression relative to sh empty. (c) Representative images of sh empty and *LAMC2* knockdown cells grown as monolayers. (d) Cell viability of sh empty and *LAMC2* knockdown cells. Cell viability was evaluated using a cell-counting-kit 8, and absorbance was measured at 450 nm. (e) Cell cycle analysis of sh empty and *LAMC2* knockdown cells. (PI incorporation). *p<0.05, **p<0.005, ***p<0.0005. *n*$\ge 3.$ Statistical significance was assessed by Student's t-test.

**Additional file 5: Figure S5.** Loss of *LAMC2* reduces stemness. (a) Flow cytometry for apoptotic cells as determined by AnnexinV/PI staining in control and *LAMC2 *knockdown cells.  (b) Flow cytometry quantification of CD44 in sh empty and *LAMC2* knockdown cells. (c) qPCR analysis of *CD133* in sh empty and *LAMC2* knockdown cells. Data are normalized to *GAPDH* and are presented as fold change in gene expression relative to sh empty. (d) Flow cytometry analysis of CD133 in sh empty and *LAMC2* knockdown cells. (e) Representative images of sh empty and *LAMC2* knockdown cells grown as spheres. (f) Sphere formation capacity of sh empty and *LAMC2* knockdown cells. P1= 1^st^ generation; P2= 2^nd^ generation. (g) Quantification of sphere size of sh empty and *LAMC2* knockdown cells. (h) Organoid formation capacity of sh empty and *LAMC2* knockdown cells. (i) Migration assay of sh empty and *LAMC2* knockdown cells. The nuclei were stained in blue (DAPI). (j) Migratory potential of sh empty and *LAMC2* knockdown cells. (k) Wound healing assay of sh empty and *LAMC2* knockdown cells. *p<0.05, **p<0.005, ***p<0.0005. *n*$\ge 3.$ Statistical significance was assessed by Student's t-test.

**Additional file 6: Figure S6. **Knockdown of *LAMC2* affects tumorigenicity. (a) Representative images from a gelatin degradation assay of sh empty and *LAMC2* knockdown cells. Nuclei were stained with Hoechst 33342 (blue), green represents actin (Alexa Fluor™ 488 Phalloidin) and red illustrates gelatin (Rodhamine). The white dashed line circles indicate the areas of degradation. (b) Degradation potential of sh empty and *LAMC2* knockdown cells. (c) qPCR analysis for EMT genes in sh empty and *LAMC2* knockdown cells. Data are normalized to *GAPDH* and are presented as fold change in gene expression relative to sh empty. (d) Western blot analysis of VIM in sh empty and *LAMC2* knockdown cells. Parallel β-ACTIN immunoblotting was performed. (e) Western blot analysis of MMP2 in sh empty and *LAMC2* knockdown cells. Parallel β-ACTIN immunoblotting was performed. (f) Number of tumors generated by the subcutaneous injection of sh empty and *LAMC2* knockdown cells. (g) Representatives images of tumors derived from sh empty and *LAMC2* knockdown cells subcutaneously injected into nude athymic mice. (h) qPCR analysis for *LAMC2*, EMT, *MMP2* and *MMP10 *gene expression in sh empty and *LAMC2* knockdown cells isolated from tumors. Data are normalized to *GAPDH* and are presented as fold change in gene expression relative to sh empty. *p<0.05, **p<0.005, ***p<0.0005. *n*$\ge 3.$ Statistical significance was assessed by Student's t-test.

**Additional file 7: Figure S7. **Generation of *LAMC2*-EGFP knock-in human PDAC cells. (a) Flow cytometry for IRFP in PDAC cells 48 hours post-nucleofection. All cytometry gates were established based on isotype controls. (b) Flow cytometry for EGFP in PDAC cells 20 days post-nucleofection. All cytometry gates were established based on isotype controls. (c) PCR gDNA specific integration analysis. The positions of primers are indicated by arrows. (d) FACS profiles showing the expression of EGFP in *LAMC2*-EGFP^‐^ and *LAMC2*-EGFP^+^ cells. (e) qPCR analysis of *LAMC2* in EGFP^+^ and EGFP^-^cells. Data are normalized to *GAPDH* and are presented as fold change in gene expression relative to the EGFP- cells. (f) Representative images of EGFP^+^ and EGFP^-^cells grown as monolayers. (g) Cell cycle analysis of EGFP^+^ and EGFP^-^cells (PI incorporation). (h) Sphere formation capacity of EGFP^+^ and EGFP^-^ cells. *p<0.05, **p<0.005, ***p<0.0005. *n*$\ge 3.$Statistical significance was assessed by Student's t-test.

**Additional file 8: Figure S8.** Characterization of human *LAMC2*-EGFP PDAC cells *in vitro *and *in vivo. *(a) Representative images of EGFP^+^ and EGFP^-^cells grown in Matrigel. (b) Organoid formation capacity of EGFP^+^ and EGFP^-^cells. (c) Quantification of organoid size of EGFP^+^ and EGFP^-^cells. (d) qPCR analysis for *LAMC2*, EMT, *MMP2* and *MMP10 *gene expression in EGFP^+^ and EGFP^-^cells. Data are normalized to *GAPDH* and are presented as fold change in gene expression relative to the EGFP^-^cells. (e) Tumor volume of EGFP^+^ and EGFP^-^ cells subcutaneously injected into nude athymic mice. n ≥ 10. (f) Representatives images of tumors derived from EGFP^+^ and EGFP^-^ cells subcutaneously injected into nude athymic mice. (g) Representative histologic sections of xenografts derived from EGFP^+^ and EGFP^-^. The tumor sections were stained for H&E and EGFP. (h) Representative flow cytometry for EGFP in subcutaneous tumors derived from injected EGFP^+^ and EGFP^-^cells. (i) qPCR analysis for *LAMC2*, EMT, *MMP2* and *MMP10*genes in EGFP^+^ and EGFP^-^cells isolated from tumors. Data are normalized to *GAPDH* and are presented as fold change in gene expression relative to the EGFP^-^cells. (j) qPCR analysis for *CDH1*in EGFP^+^ and EGFP^-^ cells isolated from tumors. Data are normalized to *GAPDH* and are presented as fold change in gene expression relative to the EGFP^-^cells. *p<0.05, **p<0.005, ***p<0.0005. *n*$\ge 3.$ Statistical *s*ignificance was assessed by Student's t-test.

**Additional file 9: Figure S9. **Global gene expression profiles of *LAMC2*-EGFP^+^ and EGFP^-^-derived tumors. Heat map of differentially expressed genes in EGFP^+^ and EGFP^-^ cells isolated from tumors.

**Additional file 10: Figure S10.** Inhibition of transforming growth factor beta (TGF-β) signaling blocks LAMC2-induced metastasis. (a) Enrichment plots for pancreatic cancer, focal adhesion, hypoxia, MAPK signaling, glycolysis and gluconeogenesis pathways in EGFP^+^ versus EGFP^-^ cells isolated by FACS from subcutaneous tumors. (b) Western blot analysis for pSMAD2 and SMAD2 in EGFP- versus EGFP+ PANC-1 and #253 cells. Parallel GAPDH immunoblotting was performed. (c) Flow cytometry quantification of EGFP and ALK5-TGFβR1 in EGFP^-^ and EGFP^+^ L3.6pl and PANC-1 cells. (d) ) Flow cytometry quantification of EGFP and CD44 in EGFP^-^ and EGFP^+^ L3.6pl and PANC-1 cells treated with 10 ng/ml of recombinant TGF-β1 (rTGF-β1). (e) Representative immunofluorescence images of LAMC2 (red) and nuclei (blue, DAPI) in PDAC cells treated with 10 ng/ml of recombinant TGF-β (rTGF-β1 and rTGF-β2). (f) Correlation between *TGFβR1*, *TGFβR2*, *TGF-β1*, *TGF-β2*, *SMAD2*, *SMAD4* and *LAMC2 *in PDAC samples from the TCGA dataset. The p value is based on Pearson’s Correlation. (g) Quantification of lung metastasis area in H&E-stained sections. *p<0.05, *n*$\ge 5.$ Statistical significance was assessed by Student's t-test.

## Data Availability

The datasets used and/or analyzed during the current study are included within the article.

## Abbreviations

Adh: Adherent
CSC: Cancer Stem Cell
Ctrl: Control
EGFP^+^: LAMC2-EGFP-clones
Gem: Gemcitabine
GSEA: Gene set enrichment analysis
IHC: Immunohistochemistry
LAMC2: Laminin γ2
MMPs: Metalloproteases
NP: Normal Pancreas
PDAC: Pancreatic ductal adenocarcinoma
Sh: Short Hairpin RNA
Sph: Spheres
TIC: Tumor-initiating cells
TGF-β: Transforming growth factor beta
TGFBR1: TGF-β receptor type 1
