# Supplementary figures and images for: LAMC2 marks a tumor-initiating cell population with an aggressive signature in pancreatic cancer
Source: J Exp Clin Cancer Res. 2022 Oct 26;41:315. doi: 10.1186/s13046-022-02516-w (PMC9609288; doi:10.1186/s13046-022-02516-w)

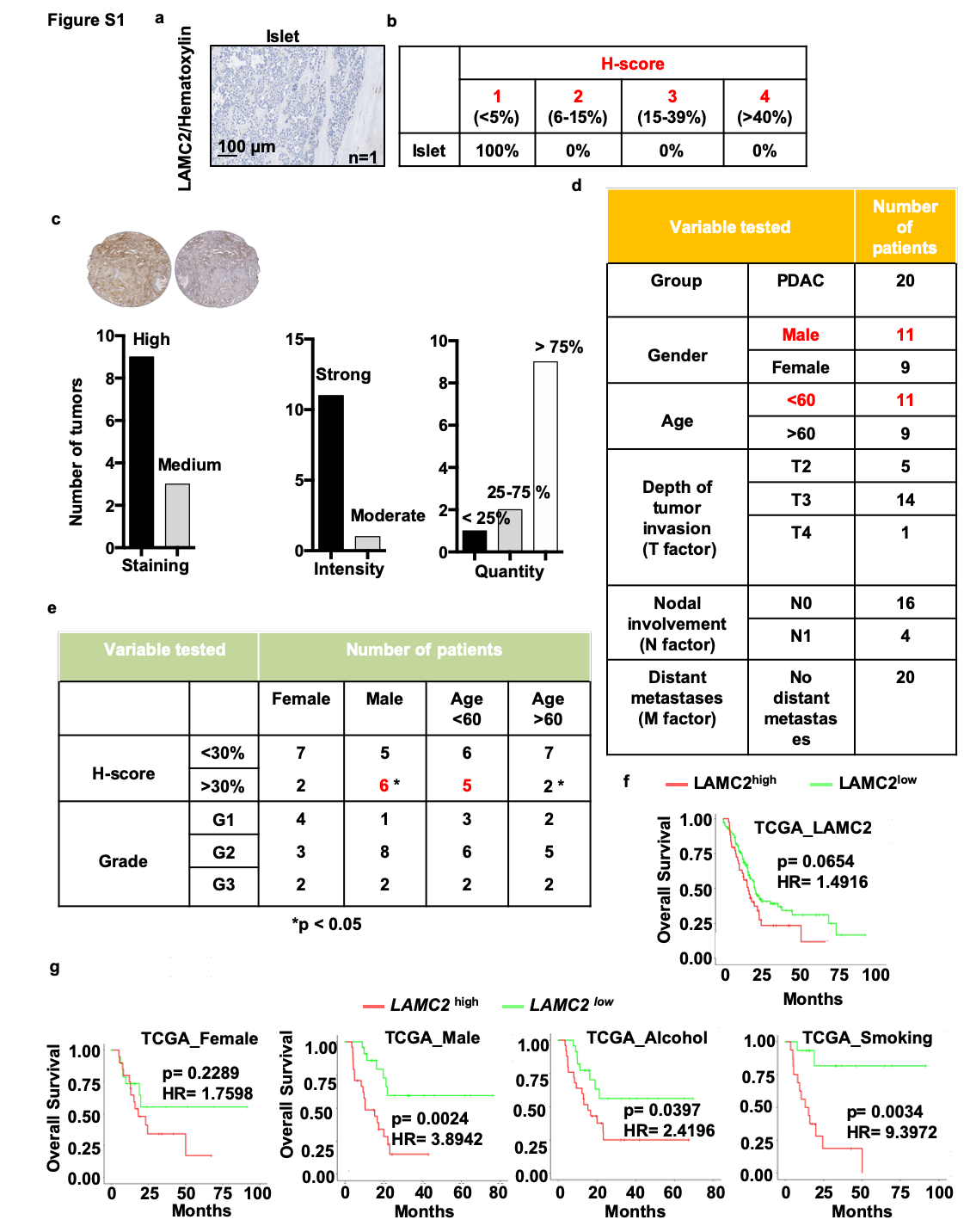

Supplement: Supplementary file 1 — Additional file 1: Figure S1. Increased LAMC2 expression is associated with an unfavorable outcome in PDAC. (a) Representative images of IHC staining for LAMC2 (brown) in tissue sections from pancreatic islets. (b) H-score for LAMC2 expression. (c) Number of PDAC tumors (Human Protein Atlas database) classified based on LAMC2 IHC staining, intensity and quantity. (d) Patients distribution according to gender, age and TNM. (e) H-score and grade distribution according to gender and age. (f) Kaplan-Meier curves showing overall survival of PDAC patients, stratified according to the median value of LAMC2 expression based on the TCGA dataset. (g) Kaplan-Meier curves showing overall survival of PDAC patients, stratified according to the median value of LAMC2 expression for gender, alcohol consumption and smoking based on the TCGA dataset. *p<0.05. Statistical significance was assessed by Student's t-test. [file 13046_2022_2516_MOESM1_ESM.docx]

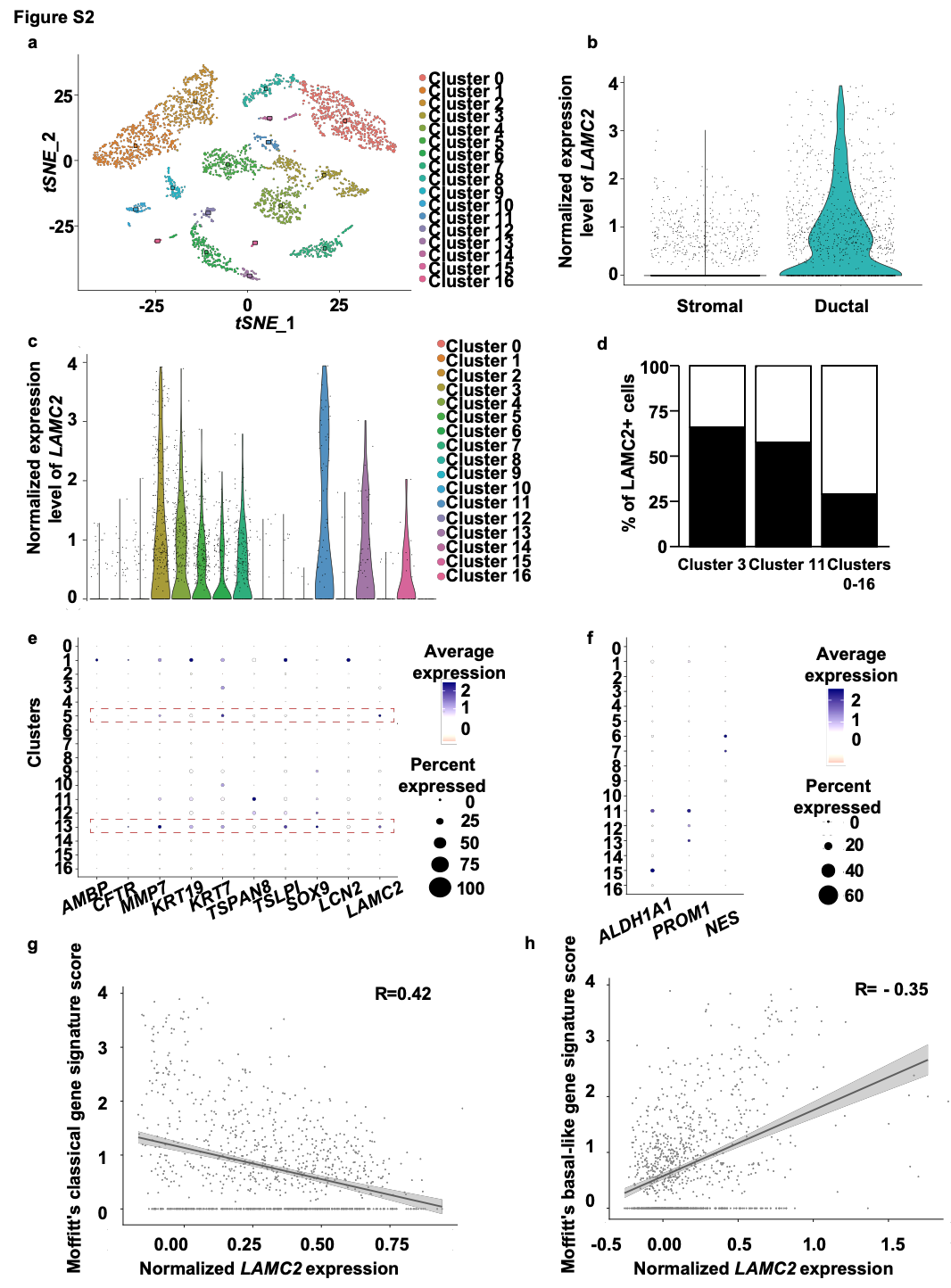

Supplement: Supplementary file 2 — Additional file 2: Figure S2. Increased LAMC2 expression is associated with aggressive signature. (a) Dimensional reduction plot (DimPlot) of multiple cell types identified in PDAC primary tumors by single-cell RNA sequencing (scRNA-Seq). The clusters are color-coded based on cell types identified using known cell type-specific markers and are visualized using t-Distributed Stochastic Neighbor embedding (t-SNE). (b) Violin plot showing expression levels of LAMC2 in stromal versus ductal (tumor) cells identified in PDAC primary tumors by scRNA-Seq. (c) Violin plot showing the expression of LAMC2 in the 17 clusters identified in PDAC primary tumors by scRNA-Seq. (d) Percentage of LAMC2 positive cells in the different clusters identified in PDAC primary tumors by scRNA-Seq. (e) DotPlot depicting expression of ductal genes in the 17 clusters identified in PDAC primary tumors by scRNA-Seq. The size of the dots represents the percentage of cells expressing the gene within a cluster, whereas the colour intensity represents the average expression level. (f) DotPlot depicting expression of stem markers genes in the 17 clusters identified in PDAC primary tumors by scRNA-Seq. The size of the dots represents the proportion of cells expressing the gene whereas the colour intensity represents the average expression level. (g) Scatter plot of LAMC2 normalized expression in the Moffitt’s classical gene signature score of PDAC. (h) Scatter plot of LAMC2 normalized expression in the Moffitt’s basal-like gene signature score of PDAC. [file 13046_2022_2516_MOESM2_ESM.docx]

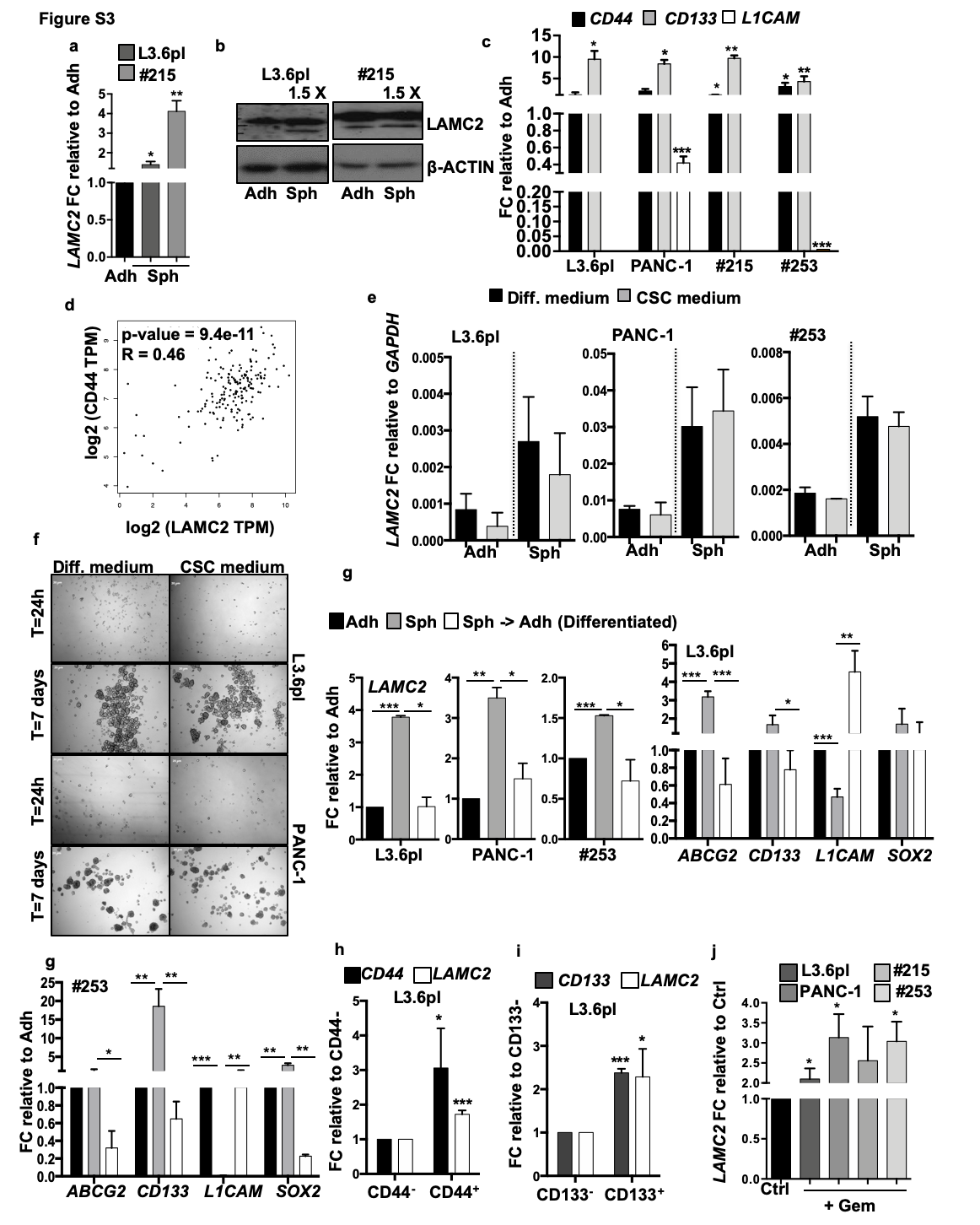

Supplement: Supplementary file 3 — Additional file 3: Figure S3. LAMC2 expression correlates with CSC content and function. (a) qPCR analysis of LAMC2 gene expression in adherent cells versus spheres. Data are normalized to GAPDH and are presented as fold change in gene expression relative to adherent cells (indicated as Adh). (b) Western blot analysis of LAMC2 in adherent cells versus spheres. Parallel β-ACTIN immunoblotting was performed. (c) qPCR analysis for CD44, CD133 and L1CAM genes in adherent cells versus spheres. Data are normalized to GAPDH and are presented as fold change in gene expression relative to adherent cells. (d) Correlation between CD44 and LAMC2 in PDAC samples from the TCGA dataset. The p value is based on Pearson’s Correlation. (e) qPCR analysis for LAMC2 in PDAC cells grown in different culture conditions. (f) qPCR analysis for LAMC2, ABCG2, CD133, L1CAM and SOX2 in Adh vs Spheres vs Differentiated cells. Data are normalized to GAPDH and are presented as fold change in gene expression relative to adherent cells. (g) Representative images of L3.6pl and PANC-1 cells growth after short (24 hours) or long (7 days) times in Diff. medium compared to CSC medium. (h) qPCR analysis for CD44 and LAMC2 genes in CD44+ sorted cells. Data are normalized to GAPDH and are presented as fold change in gene expression relative to CD44-cells. (i) qPCR analysis for CD133 and LAMC2 genes in CD133+ sorted cells. Data are normalized to GAPDH and are presented as fold change in gene expression relative to CD133- cells. (j) qPCR analysis for LAMC2 in PDAC cells treated with 100 µM of gemcitabine. Data are normalized to GAPDH and are presented as fold change in gene expression relative to control cells (untreated). *p<0.05, **p<0.005, ***p<0.0005. n$\ge 3.$≥3. S [file 13046_2022_2516_MOESM3_ESM.docx]

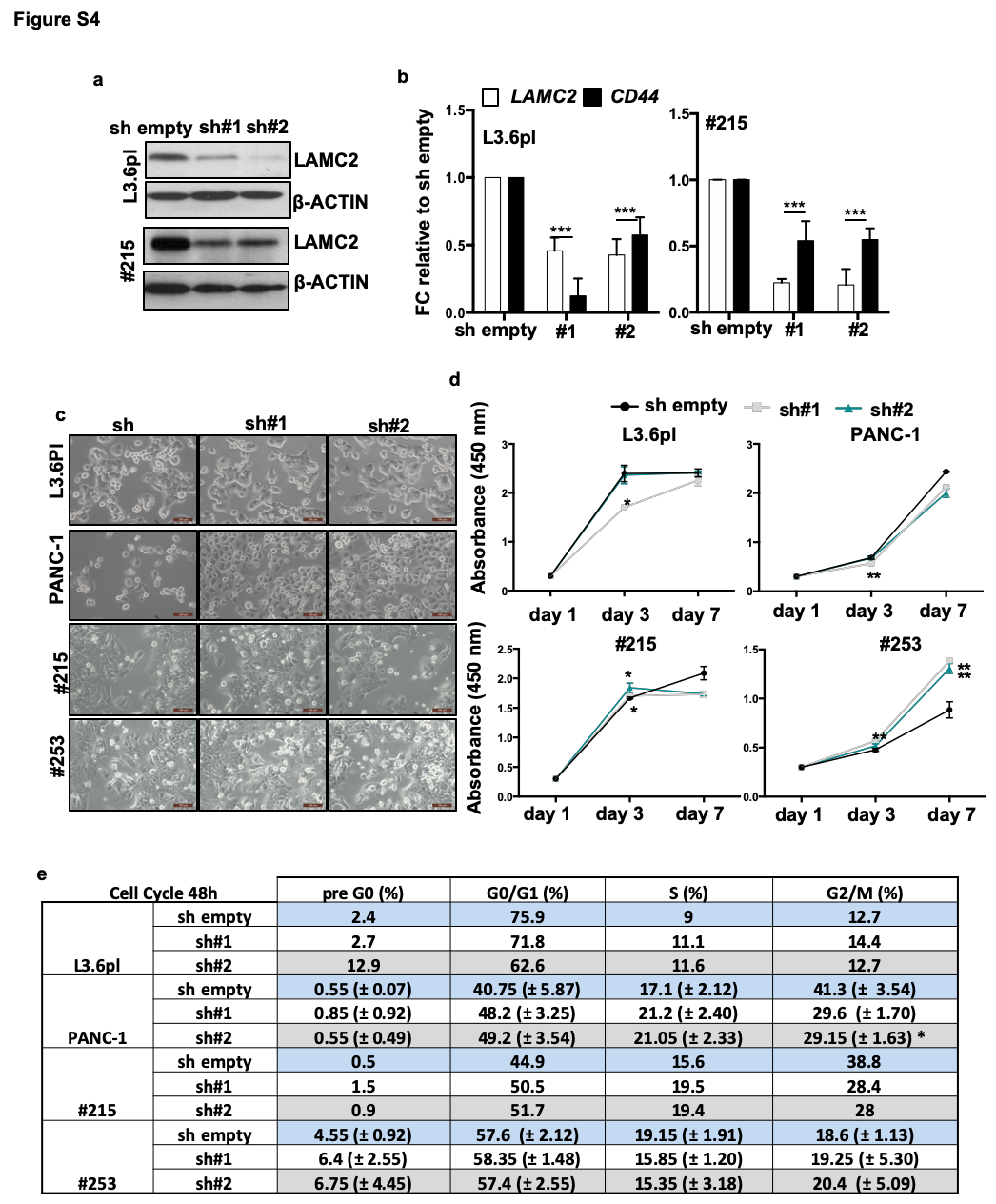

Supplement: Supplementary file 4 — Additional file 4: Figure S4. Knockdown of LAMC2 does not affect cell growth. (a) Western blot analysis of LAMC2 in sh empty and LAMC2 knockdown cells. Parallel β-ACTIN immunoblotting was performed. (b) qPCR analysis for LAMC2 and CD44 gene expression in sh empty and LAMC2 knockdown cells. Data are normalized to GAPDH and are presented as fold change in gene expression relative to sh empty. (c) Representative images of sh empty and LAMC2 knockdown cells grown as monolayers. (d) Cell viability of sh empty and LAMC2 knockdown cells. Cell viability was evaluated using a cell-counting-kit 8, and absorbance was measured at 450 nm. (e) Cell cycle analysis of sh empty and LAMC2 knockdown cells. (PI incorporation). *p<0.05, **p<0.005, ***p<0.0005. n$\ge 3.$≥3. Statistical significance was assessed by Student's t-test. [file 13046_2022_2516_MOESM4_ESM.docx]

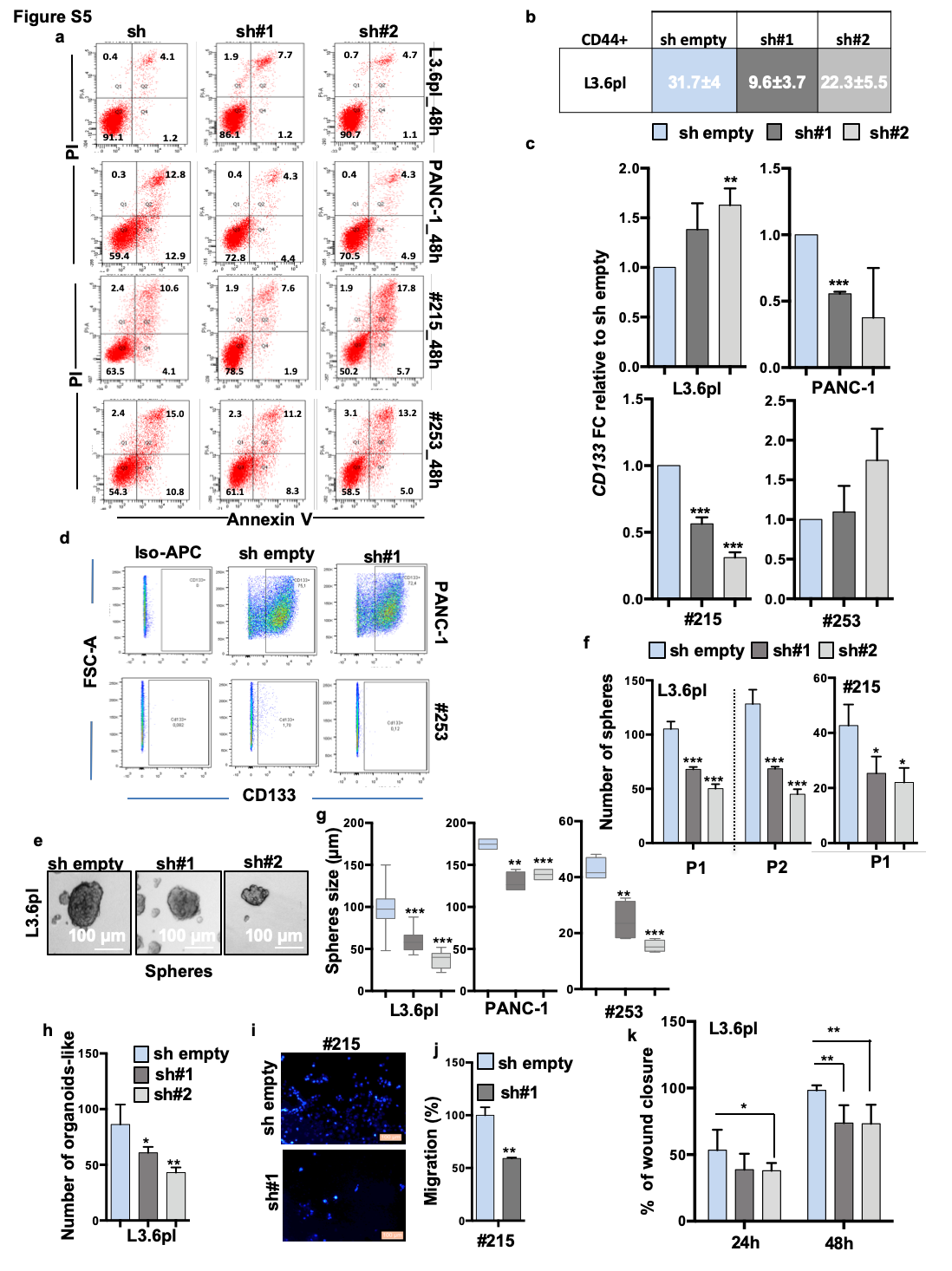

Supplement: Supplementary file 5 — Additional file 5: Figure S5. Loss of LAMC2 reduces stemness. (a) Flow cytometry for apoptotic cells as determined by AnnexinV/PI staining in control and LAMC2 knockdown cells. (b) Flow cytometry quantification of CD44 in sh empty and LAMC2 knockdown cells. (c) qPCR analysis of CD133 in sh empty and LAMC2 knockdown cells. Data are normalized to GAPDH and are presented as fold change in gene expression relative to sh empty. (d) Flow cytometry analysis of CD133 in sh empty and LAMC2 knockdown cells. (e) Representative images of sh empty and LAMC2 knockdown cells grown as spheres. (f) Sphere formation capacity of sh empty and LAMC2 knockdown cells. P1= 1st generation; P2= 2nd generation. (g) Quantification of sphere size of sh empty and LAMC2 knockdown cells. (h) Organoid formation capacity of sh empty and LAMC2 knockdown cells. (i) Migration assay of sh empty and LAMC2 knockdown cells. The nuclei were stained in blue (DAPI). (j) Migratory potential of sh empty and LAMC2 knockdown cells. (k) Wound healing assay of sh empty and LAMC2 knockdown cells. *p<0.05, **p<0.005, ***p<0.0005. n$\ge 3.$≥3. Statistical significance was assessed by Student's t-test. [file 13046_2022_2516_MOESM5_ESM.docx]

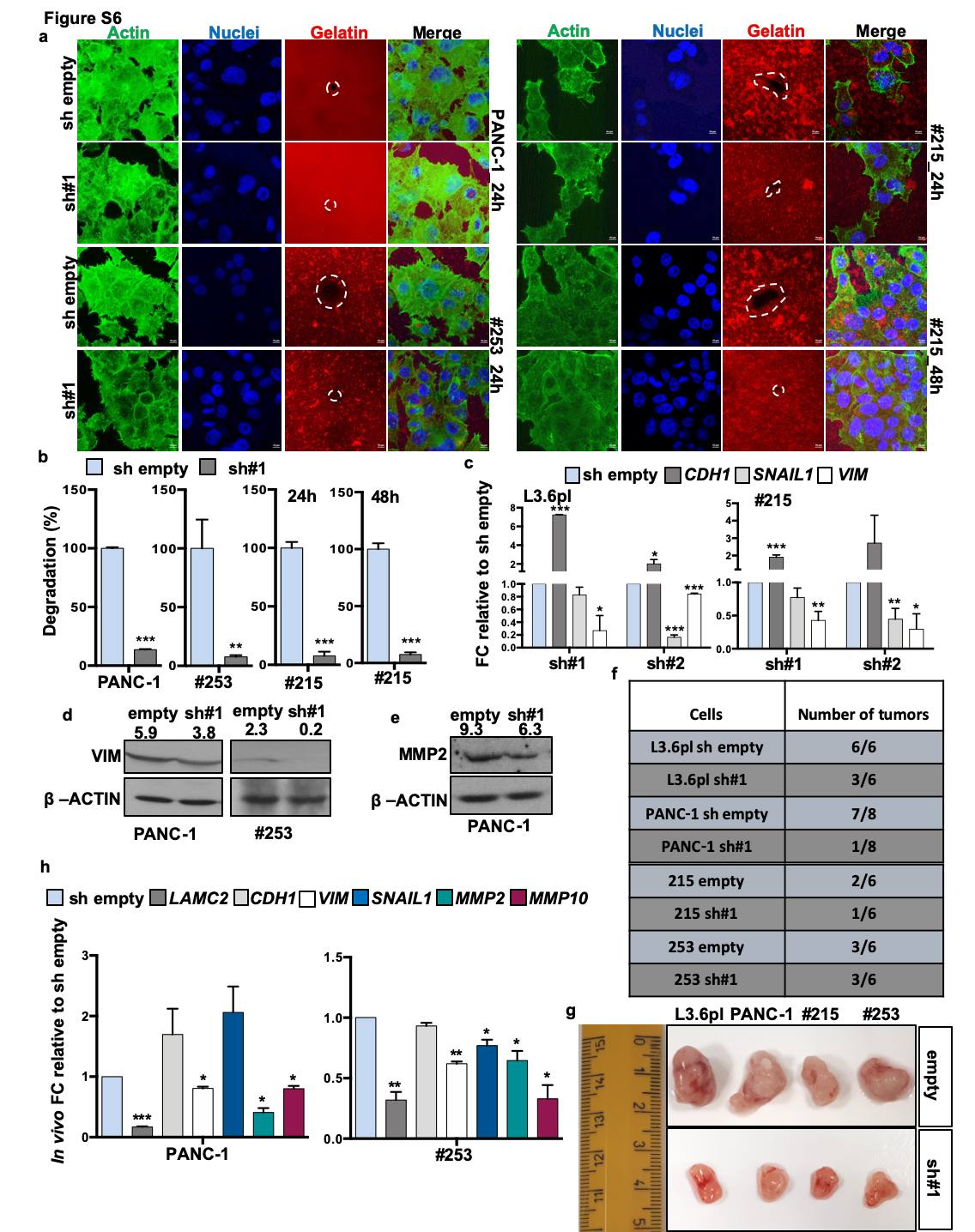

Supplement: Supplementary file 6 — Additional file 6: Figure S6. Knockdown of LAMC2 affects tumorigenicity. (a) Representative images from a gelatin degradation assay of sh empty and LAMC2 knockdown cells. Nuclei were stained with Hoechst 33342 (blue), green represents actin (Alexa Fluor™ 488 Phalloidin) and red illustrates gelatin (Rodhamine). The white dashed line circles indicate the areas of degradation. (b) Degradation potential of sh empty and LAMC2 knockdown cells. (c) qPCR analysis for EMT genes in sh empty and LAMC2 knockdown cells. Data are normalized to GAPDH and are presented as fold change in gene expression relative to sh empty. (d) Western blot analysis of VIM in sh empty and LAMC2 knockdown cells. Parallel β-ACTIN immunoblotting was performed. (e) Western blot analysis of MMP2 in sh empty and LAMC2 knockdown cells. Parallel β-ACTIN immunoblotting was performed. (f) Number of tumors generated by the subcutaneous injection of sh empty and LAMC2 knockdown cells. (g) Representatives images of tumors derived from sh empty and LAMC2 knockdown cells subcutaneously injected into nude athymic mice. (h) qPCR analysis for LAMC2, EMT, MMP2 and MMP10 gene expression in sh empty and LAMC2 knockdown cells isolated from tumors. Data are normalized to GAPDH and are presented as fold change in gene expression relative to sh empty. *p<0.05, **p<0.005, ***p<0.0005. n$\ge 3.$≥3. Statistical significance was assessed by Student's t-test. [file 13046_2022_2516_MOESM6_ESM.docx]

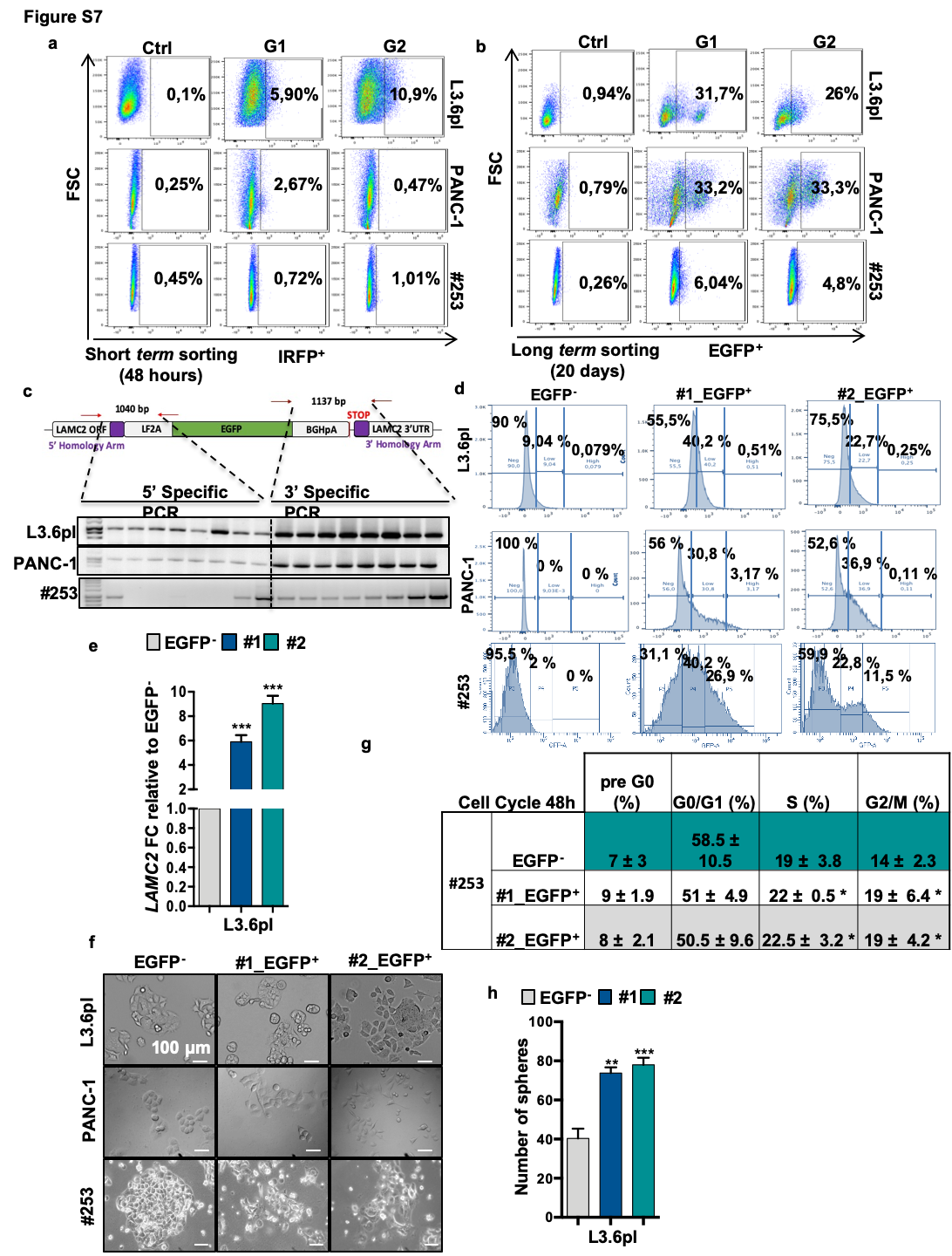

Supplement: Supplementary file 7 — Additional file 7: Figure S7. Generation of LAMC2-EGFP knock-in human PDAC cells. (a) Flow cytometry for IRFP in PDAC cells 48 hours post-nucleofection. All cytometry gates were established based on isotype controls. (b) Flow cytometry for EGFP in PDAC cells 20 days post-nucleofection. All cytometry gates were established based on isotype controls. (c) PCR gDNA specific integration analysis. The positions of primers are indicated by arrows. (d) FACS profiles showing the expression of EGFP in LAMC2-EGFP‐ and LAMC2-EGFP+ cells. (e) qPCR analysis of LAMC2 in EGFP+ and EGFP-cells. Data are normalized to GAPDH and are presented as fold change in gene expression relative to the EGFP- cells. (f) Representative images of EGFP+ and EGFP-cells grown as monolayers. (g) Cell cycle analysis of EGFP+ and EGFP-cells (PI incorporation). (h) Sphere formation capacity of EGFP+ and EGFP- cells. *p<0.05, **p<0.005, ***p<0.0005. n$\ge 3.$≥3.Statistical significance was assessed by Student's t-test. [file 13046_2022_2516_MOESM7_ESM.docx]

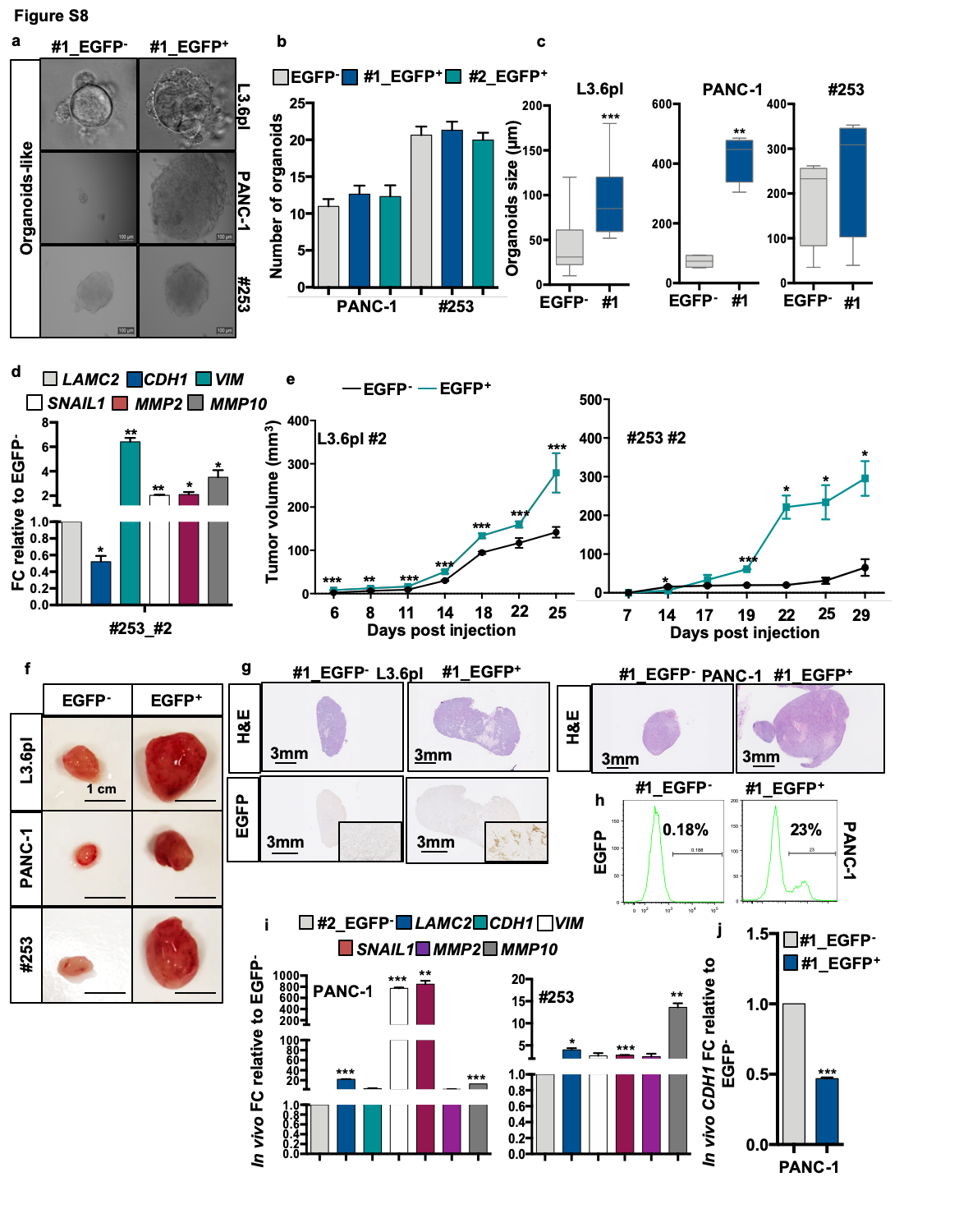

Supplement: Supplementary file 8 — Additional file 8: Figure S8. Characterization of human LAMC2-EGFP PDAC cells in vitro and in vivo. (a) Representative images of EGFP+ and EGFP-cells grown in Matrigel. (b) Organoid formation capacity of EGFP+ and EGFP-cells. (c) Quantification of organoid size of EGFP+ and EGFP-cells. (d) qPCR analysis for LAMC2, EMT, MMP2 and MMP10 gene expression in EGFP+ and EGFP-cells. Data are normalized to GAPDH and are presented as fold change in gene expression relative to the EGFP-cells. (e) Tumor volume of EGFP+ and EGFP- cells subcutaneously injected into nude athymic mice. n ≥ 10. (f) Representatives images of tumors derived from EGFP+ and EGFP- cells subcutaneously injected into nude athymic mice. (g) Representative histologic sections of xenografts derived from EGFP+ and EGFP-. The tumor sections were stained for H&E and EGFP. (h) Representative flow cytometry for EGFP in subcutaneous tumors derived from injected EGFP+ and EGFP-cells. (i) qPCR analysis for LAMC2, EMT, MMP2 and MMP10genes in EGFP+ and EGFP-cells isolated from tumors. Data are normalized to GAPDH and are presented as fold change in gene expression relative to the EGFP-cells. (j) qPCR analysis for CDH1in EGFP+ and EGFP- cells isolated from tumors. Data are normalized to GAPDH and are presented as fold change in gene expression relative to the EGFP-cells. *p<0.05, **p<0.005, ***p<0.0005. n$\ge 3.$≥3. Statistical significance was assessed by Student's t-test. [file 13046_2022_2516_MOESM8_ESM.docx]

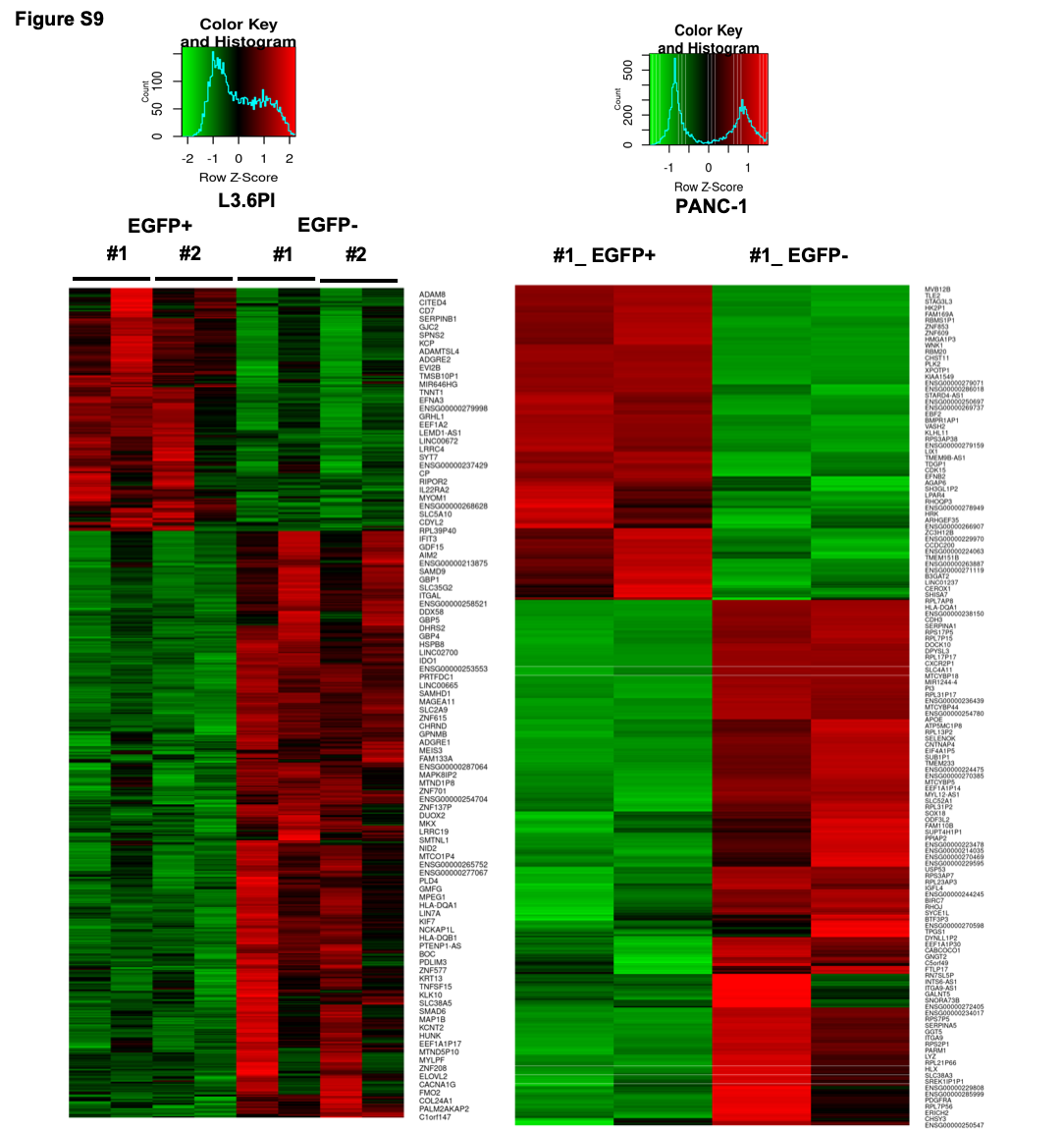

Supplement: Supplementary file 9 — Additional file 9: Figure S9. Global gene expression profiles of LAMC2-EGFP+ and EGFP--derived tumors. Heat map of differentially expressed genes in EGFP+ and EGFP- cells isolated from tumors. [file 13046_2022_2516_MOESM9_ESM.docx]

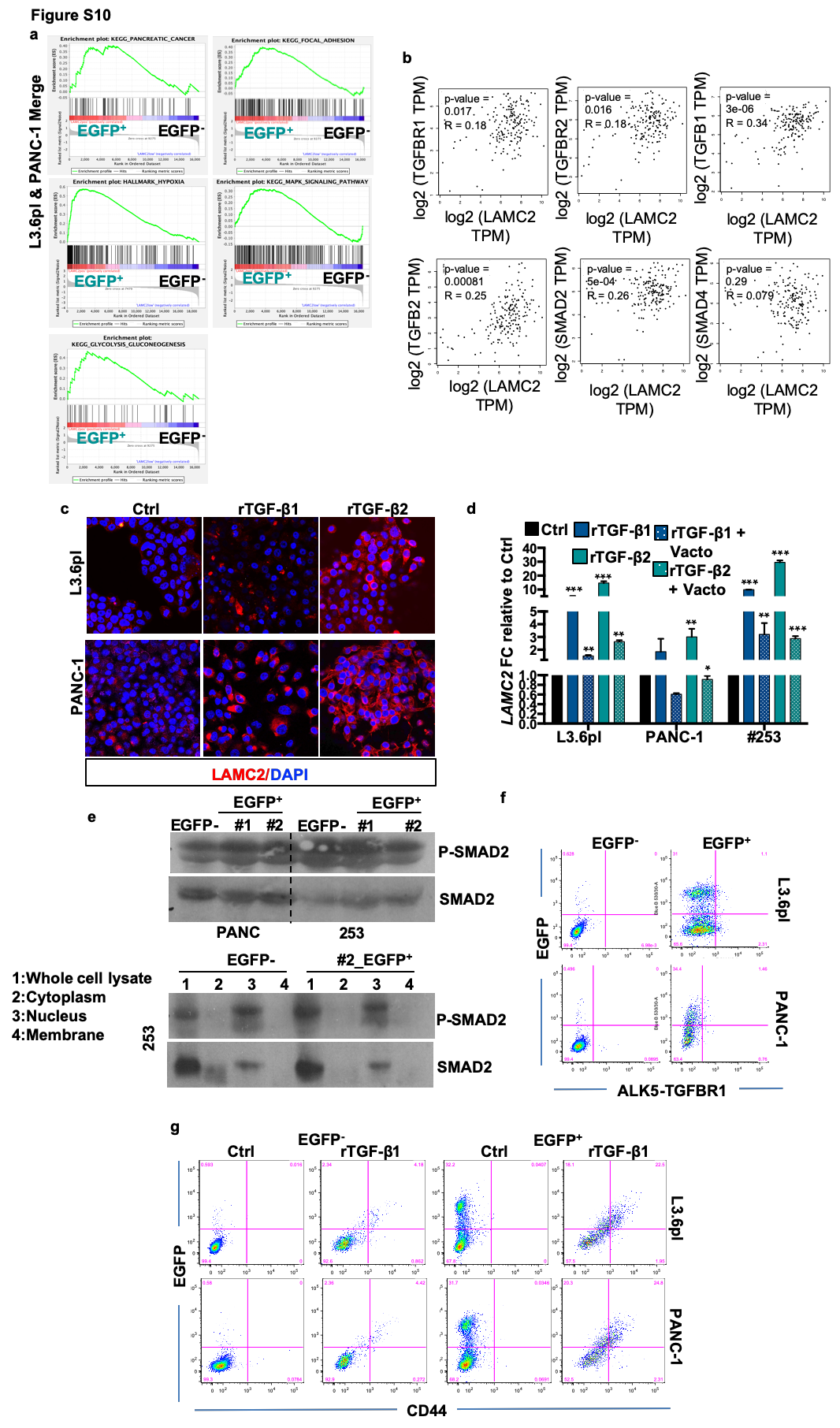

Supplement: Supplementary file 10 — Additional file 10: Figure S10. Inhibition of transforming growth factor beta (TGF-β) signaling blocks LAMC2-induced metastasis. (a) Enrichment plots for pancreatic cancer, focal adhesion, hypoxia, MAPK signaling, glycolysis and gluconeogenesis pathways in EGFP+ versus EGFP- cells isolated by FACS from subcutaneous tumors. (b) Western blot analysis for pSMAD2 and SMAD2 in EGFP- versus EGFP+ PANC-1 and #253 cells. Parallel GAPDH immunoblotting was performed. (c) Flow cytometry quantification of EGFP and ALK5-TGFβR1 in EGFP- and EGFP+ L3.6pl and PANC-1 cells. (d) ) Flow cytometry quantification of EGFP and CD44 in EGFP- and EGFP+ L3.6pl and PANC-1 cells treated with 10 ng/ml of recombinant TGF-β1 (rTGF-β1). (e) Representative immunofluorescence images of LAMC2 (red) and nuclei (blue, DAPI) in PDAC cells treated with 10 ng/ml of recombinant TGF-β (rTGF-β1 and rTGF-β2). (f) Correlation between TGFβR1, TGFβR2, TGF-β1, TGF-β2, SMAD2, SMAD4 and LAMC2 in PDAC samples from the TCGA dataset. The p value is based on Pearson’s Correlation. (g) Quantification of lung metastasis area in H&E-stained sections. *p<0.05, n$\ge 5.$≥5. Statistical significance was assessed by Student's t-test. [file 13046_2022_2516_MOESM10_ESM.docx]
